# Oxidative stress via inhibition of the mitochondrial electron transport and Nrf-2-mediated anti-oxidative response regulate the cytotoxic activity of plumbagin

**DOI:** 10.1038/s41598-018-19261-w

**Published:** 2018-01-18

**Authors:** Arvinder Kapur, Thomas Beres, Kavya Rathi, Amruta P. Nayak, Austin Czarnecki, Mildred Felder, Amani Gillette, Spencer S. Ericksen, Emmanuel Sampene, Melissa C. Skala, Lisa Barroilhet, Manish S. Patankar

**Affiliations:** 1 Department of Obstetrics and Gynecology, University of Wisconsin-Madison, Madison, WI 53792-6188 USA; 2 Indian Institute for Science Education and Research, Pune, India; 3 Morgridge Institute for Research and the Department of Biomedical Engineering, University of Wisconsin-Madison, Madison, WI USA; 4 Small Molecule Screening Facility, University of Wisconsin Paul P. Carbone Comprehensive Cancer Center, School of Medicine and Public Health, University of Wisconsin-Madison, Madison, WI USA; 5 Department of Biostatistics, University of Wisconsin-Madison, Madison, WI USA

## Abstract

Plumbagin, an anti-cancer agent, is toxic to cells of multiple species. We investigated if plumbagin targets conserved biochemical processes. Plumbagin induced DNA damage and apoptosis in cells of diverse mutational background with comparable potency. A 3–5 fold increase in intracellular oxygen radicals occurred in response to plumbagin. Neutralization of the reactive oxygen species by N-acetylcysteine blocked apoptosis, indicating a central role for oxidative stress in plumbagin-mediated cell death. Plumbagin docks in the ubiquinone binding sites (Q_0_ and Q_i_) of mitochondrial complexes I–III, the major sites for oxygen radicals. Plumbagin decreased oxygen consumption rate, ATP production and optical redox ratio (NAD(P)H/FAD) indicating interference with electron transport downstream of mitochondrial Complex II. Oxidative stress induced by plumbagin triggered an anti-oxidative response via activation of Nrf2. Plumbagin and the Nrf2 inhibitor, brusatol, synergized to inhibit cell proliferation. These data indicate that while inhibition of electron transport is the conserved mechanism responsible for plumbagin’s chemotoxicity, activation of Nrf2 is the resulting anti-oxidative response that allows plumbagin to serve as a chemopreventive agent. This study provides the basis for designing potent and selective plumbagin analogs that can be coupled with suitable Nrf2 inhibitors for chemotherapy or administered as single agents to induce Nrf2-mediated chemoprevention.

## Introduction

Plumbagin, a naturally occurring 1,4-naphthoquinone, is a potent inducer of cytotoxicity in prostate, pancreatic, breast and lung cancer cells^1–5^. Plumbagin treatment also delays the onset of cancer in a transgenic mouse model for prostate cancer^5^.

Plumbagin produces its anti-cancer effects by inducing apoptosis and G1 cell cycle arrest^3^. Evidence supporting these observations includes an increase in Annexin V staining, cleaved caspase-3 activity, and expression of Bax and a corresponding decrease in the anti-apoptotic protein, Bcl-2. Cell cycle arrest in plumbagin-treated non-small cell lung cancer cells correlates with inhibition of Cyclin D1 and Cyclin E^3^. While these downstream effects are consistent in many of the reports describing the cytotoxic activity of plumbagin, the upstream events that lead to apoptosis are unclear.

As is the case with many natural products, plumbagin modulates a variety of cell signaling mechanisms that may independently trigger apoptosis. Some of the prominent observations are the increase in expression of p53 and reduction in activation of NFκB and survivin^3,4^. Decreased expression of PKCε, Cox-2, and Stat3 in a prostate cancer model have important roles in inducing apoptosis in plumbagin-treated cancer cells^5^. Finally, several reports have indicated that exposure to plumbagin causes an increase in intracellular oxygen radicals^6,7^. This spike in reactive oxygen species (ROS) causes double strand DNA breaks and likely contributes to cell death^6,8^. Based on this evidence, plumbagin, its chemical analogs and its complexes in nanoparticles and chitosan microspheres are being considered as potential chemotherapeutic and chemopreventive agents.

An intriguing aspect of plumbagin is that this compound is able to inhibit cancer cells that have diverse mutational status and tissue of origin. Additionally, not only is plumbagin effective against cancer cells but also is being investigated for the treatment of fulminant hepatic failure and bacterial, fungal and helminthic infections^9^.

Because plumbagin’s cytotoxic activity is demonstrated in multiple types of cancer, mammalian cells, bacteria and unicellular and multicellular parasites we asked if plumbagin was targeting an evolutionarily conserved pathway/biochemical process that was critical to the survival of organisms. If this hypothesis is correct, then understanding this evolutionarily conserved mechanism will be important for the further development of plumbagin and its analogs as anti-cancer agents. With that objective, we conducted studies on the early cellular events responsible for the cytotoxic activity of plumbagin in cancer cells. Here, we present data that oxidative stress mediated by plumbagin is a primary cellular insult essential for its cytotoxicity. The increase in intracellular oxygen radicals observed in cancer cells occurs because plumbagin is able to interfere with mitochondrial electron transport because of its close structural relationship with ubiquinone (Coenzyme Q, CoQ), resulting in decreased oxygen consumption and generation of oxygen radicals. The current study provides important information for the development of chemotherapeutic plumbagin analogs that are more potent and selective in targeting tumors.

## Results

### Plumbagin reduces the viability of an array of cancer cell lines

**Figure 1:**
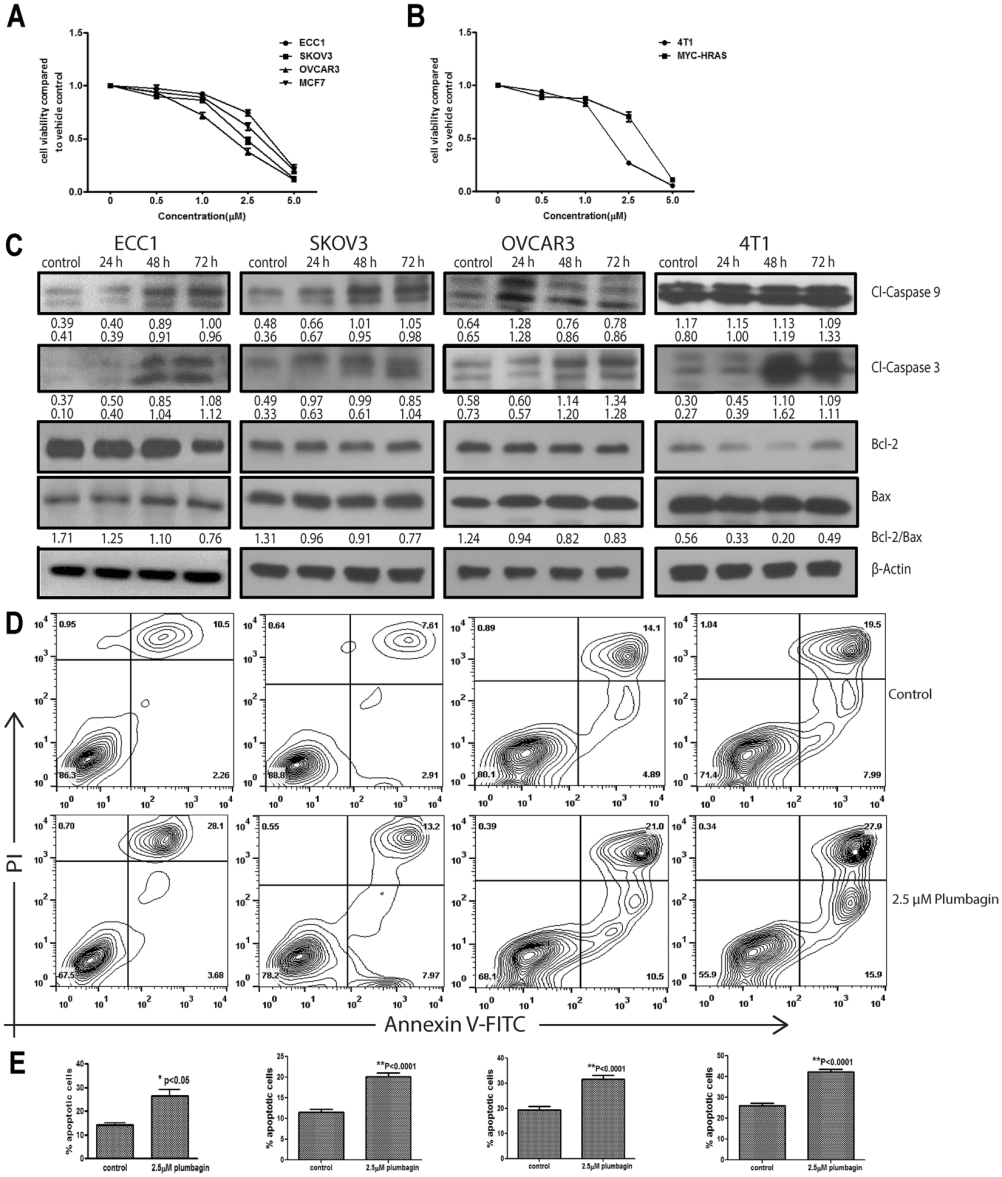
Plumbagin inhibits proliferation and induces apoptosis in cancer cells. A panel of human (**A**) and murine (**B**) cancer cell lines were treated with plumbagin (0–5 μM) for 72 hours and proliferation of the cell lines was determined using MTT assay. The assays were conducted in triplicate (biological replicates) and in each experiment, there were eight technical replicates of each condition tested. The plots in A and B are average data from the three biological replicates for each cell line. Decreased proliferation in plumbagin (2.5 μM) treated ECC1, SKOV3, OVCAR3 and 4T1 cells was due to apoptosis as determined by the increase in cleaved caspase 9 (Cl-Caspase 9) and cleaved caspase 3 (Cl-Caspase 3) and decrease in Bcl-2/Bax ratio as determined by western blotting (**C**). The numerical values under each blot are densitometry data for the bands. The two values under the caspase blots are for the two bands of the proteins that are normalized to b-actin (bottom panel). The densitometry values under the Bax blot shows ratio of Bcl2/Bax. The full blots are presented in Supplementary File 10. Apoptosis in ECC1, SKOV3, OVCAR3 and 4T1 cells treated for 24 h with 2.5 μM plumbagin was also confirmed by staining with FITC-conjugated Annexin V and propidium iodide. The percentage of cells undergoing apoptosis was determined by flow cytometry. Contour plots show cell populations that are Annexin V^pos^ and those that are Annexin V and propidium iodide double positive (**D**).The average flow cytometry results from three biological replicates conducted with each of the four cell lines are summarized in the bar graph in (**E**).

First, we confirmed broad spectrum activity of plumbagin by testing its effects on the proliferation of human (ECC1, SKOV3, OVCAR3 and MCF7) and murine (4T1 and MYC-HRAS MOSE) cancer cell lines. OVCAR3, SKOV3, MCF7, and ECC1 are human ovarian, breast and endometrial cancer cells. 4T1 is a cell line derived from a spontaneous mammary tumor from BALB/c mouse and the MYC-HRAS MOSE are murine ovarian surface epithelial cells transformed by the introduction of mutant MYC and HRAS. Irrespective of their mutational status or tissue and species of origin, 3-(4,5-dimethythiazol-2-yl)-2,5-diphenyl tetrazolium bromide (MTT) assays conducted with all of these cells showed that plumbagin was effective in inhibiting their proliferation at IC_50_ between 1.5–3.5 μM (Fig. 1A and B; Supplementary Files 1 and 2).

### Plumbagin causes apoptosis

The decrease in cell viability of plumbagin-treated ECC1, SKOV3, OVCAR3 and 4T1 cells was due to induction of apoptosis as measured by an increase in cleaved caspase-9 and caspase-3 (Fig. 1C) and staining with Annexin V and propidium iodide (Fig. 1D and E). In all four cell lines tested, decrease in Bcl-2/BAX ratio was noted after exposure to plumbagin for 24 h, 48 h and 72 h (Fig. 1C).

### Oxidative stress induced by plumbagin is essential for apoptosis

**Figure 2:**
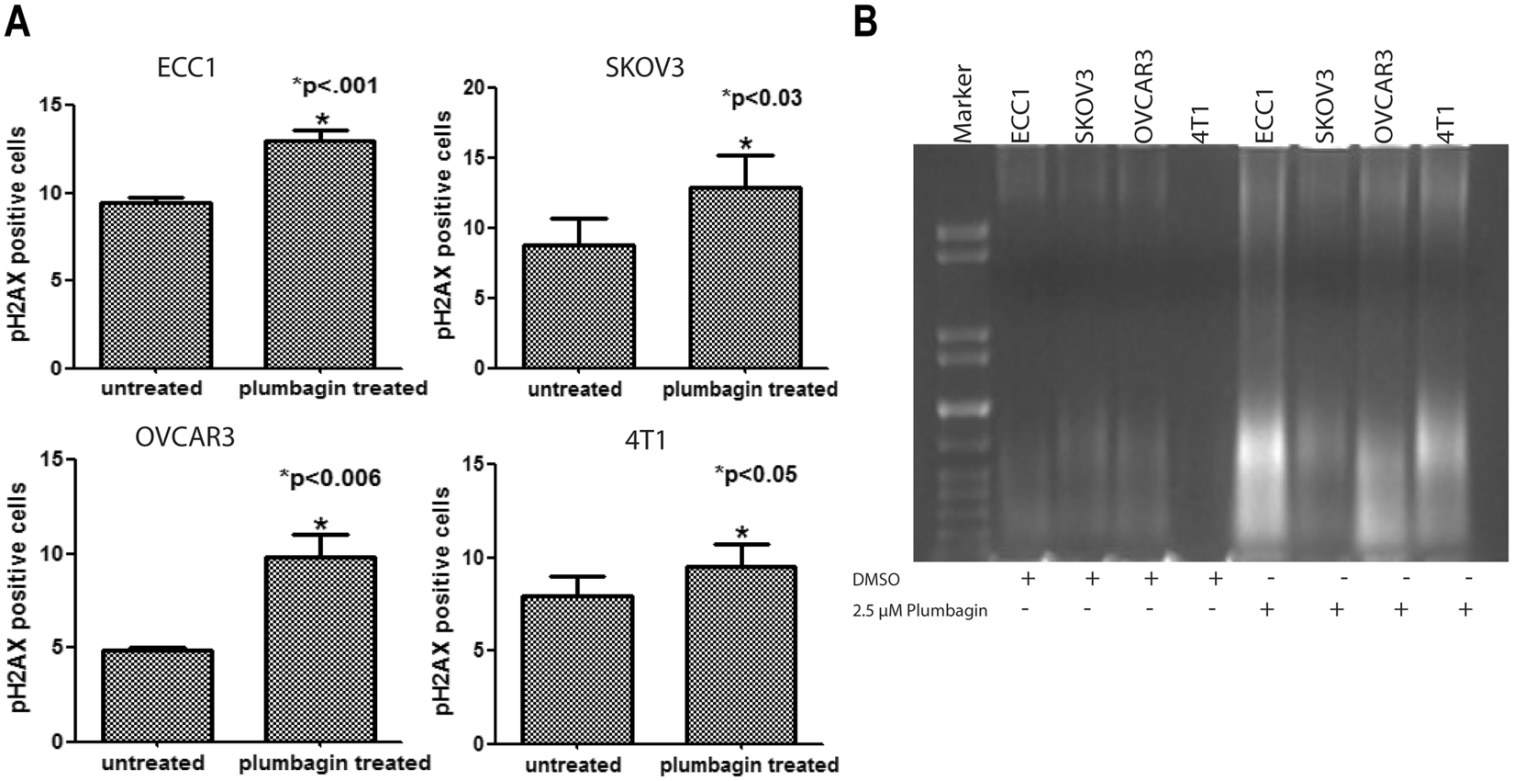
Treatment with plumbagin induces DNA damage in cancer cells. DNA damage in ECC1, SKOV3, OVCAR3 and 4T1 cells treated with plumbagin (2.5 mM) for 24 h was assessed by monitoring phospho-H2AX (pH2AX) (**A**) and DNA degradation (**B**). DMSO was used as the vehicle control in all experiments. pH2AX was measured by flow cytometry. Average pH2AX levels from three biological replicates are shown in A. The blot in B is representative of three biological replicates.

**Figure 3:**
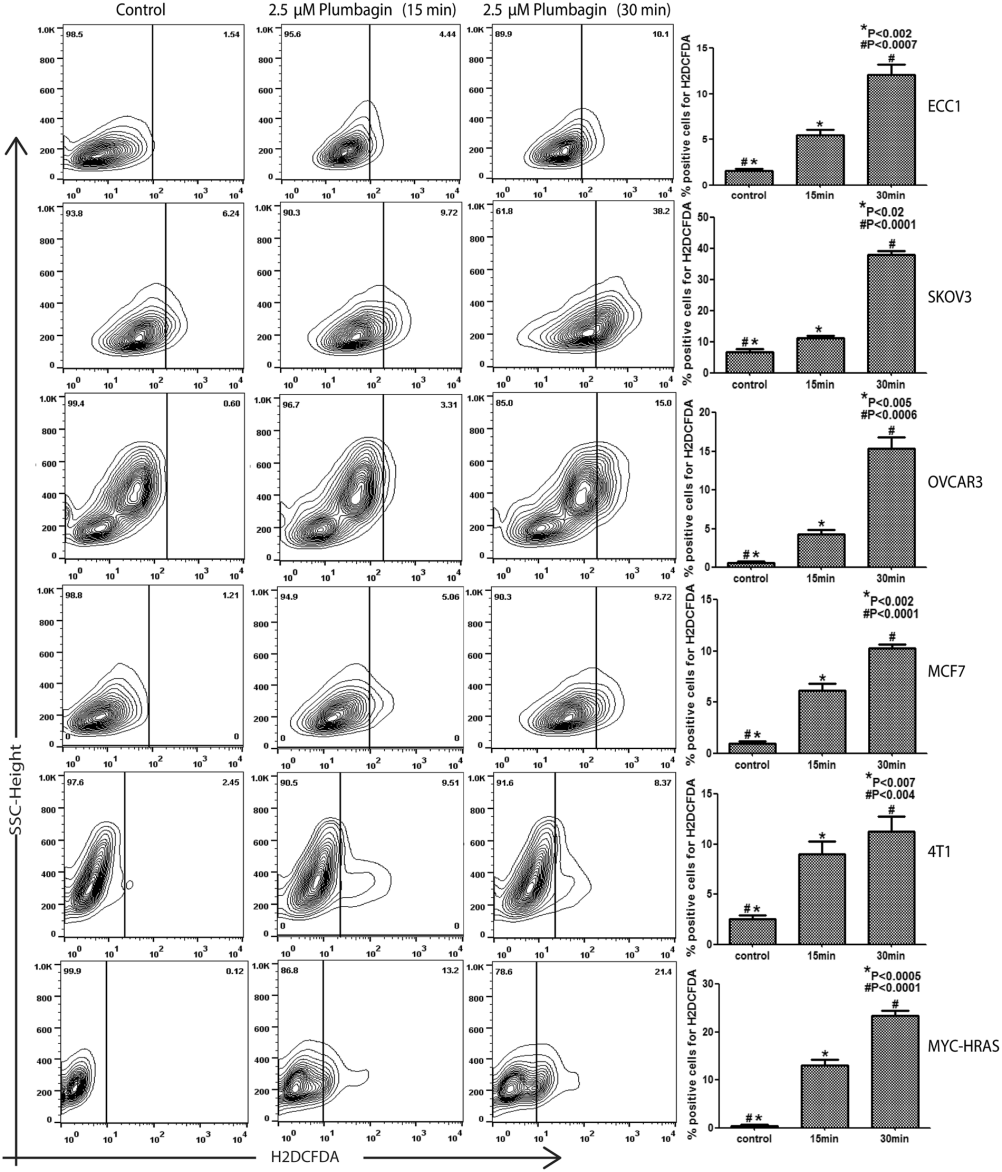
Plumbagin induces oxidative stress in cancer cells. ECC1, SKOV3, OVCAR3, MCF7, 4T1 and MYC-HRAS cells pre-labeled with the oxygen radical-sensing dye H2CFDA were treated with plumbagin (2.5 μM) for 15 and 30 min. The increase in intracellular oxygen radicals was determined by flow cytometry. Contour plots shown are representative of three biological replicates. The bar graphs provide average of the fluorescence levels from the three biological replicates for each cell line.

Apoptosis observed in plumbagin treated cells was associated with an increase in phosphoH2AX (pH2AX) staining as well as DNA damage in all cell lines tested (Fig. 2A,B). These observations coupled with previous reports^6,8^ suggested that plumbagin was likely inducing oxidative stress in the cancer cells. Potentially, the oxidative stress induced by plumbagin could result in apoptosis of the cancer cells. Support for these hypotheses was obtained from our observations that plumbagin treatment caused a rapid increase in intracellular oxygen radicals in ECC1, SKOV3, OVCAR3, MCF7, 4T1 and Myc-HRAS cells treated with plumbagin (Fig. 3).

**Figure 4:**
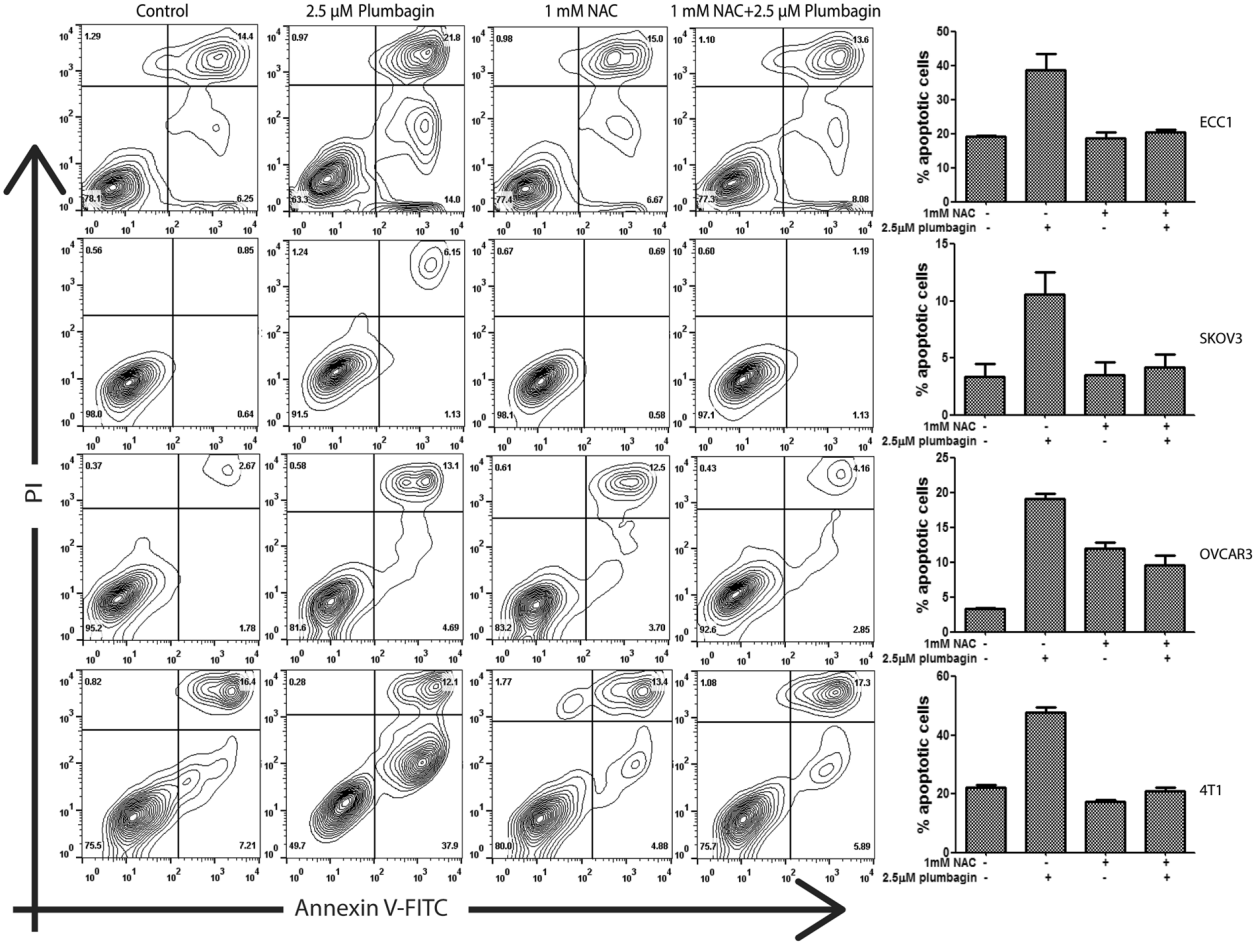
Inhibition of oxidative stress attenuates plumbagin-induced apoptosis. ECC1, SKOV3, OVCAR3 and 4T1 cells were pre-treated with 1 mM NAC. After subsequent treatment with plumbagin (2.5 µM for 24 h) cells were labeled with Annexin-V-FITC and propidium iodide. Apoptosis was monitored by flow cytometry. Contour plots shown are representative of three biological replicates. The bar graphs provide average of the fluorescence levels from the three biological replicates for each cell line. P-values for the bar chart are provided in Supplementary File 3.

To prove that oxidative stress was responsible for plumbagin’s ability to decrease the viability of cancer cells, we pre-treated ECC1 cells with the oxygen radical scavenger N-acetylcysteine (NAC) for 30 min. Subsequently, NAC was removed and cells were washed. This step was taken to eliminate NAC in the media that potentially would reduce plumbagin by reacting to its naphthoquinone reactive center. After removal of excess NAC, the ECC1, SKOV3, OVCAR3 and 4T1 cells were treated with plumbagin for 24 h and viability of the cells was monitored. Pre-treatment of the cells with NAC significantly increased the viability and decreased apoptosis (as indicated by Annexin V staining) of plumbagin-treated cells (Fig. 4; Supplementary File 3).

### Plumbagin docks to the ubiquinone binding site of mitochondrial complexes I and III

**Figure 5:**
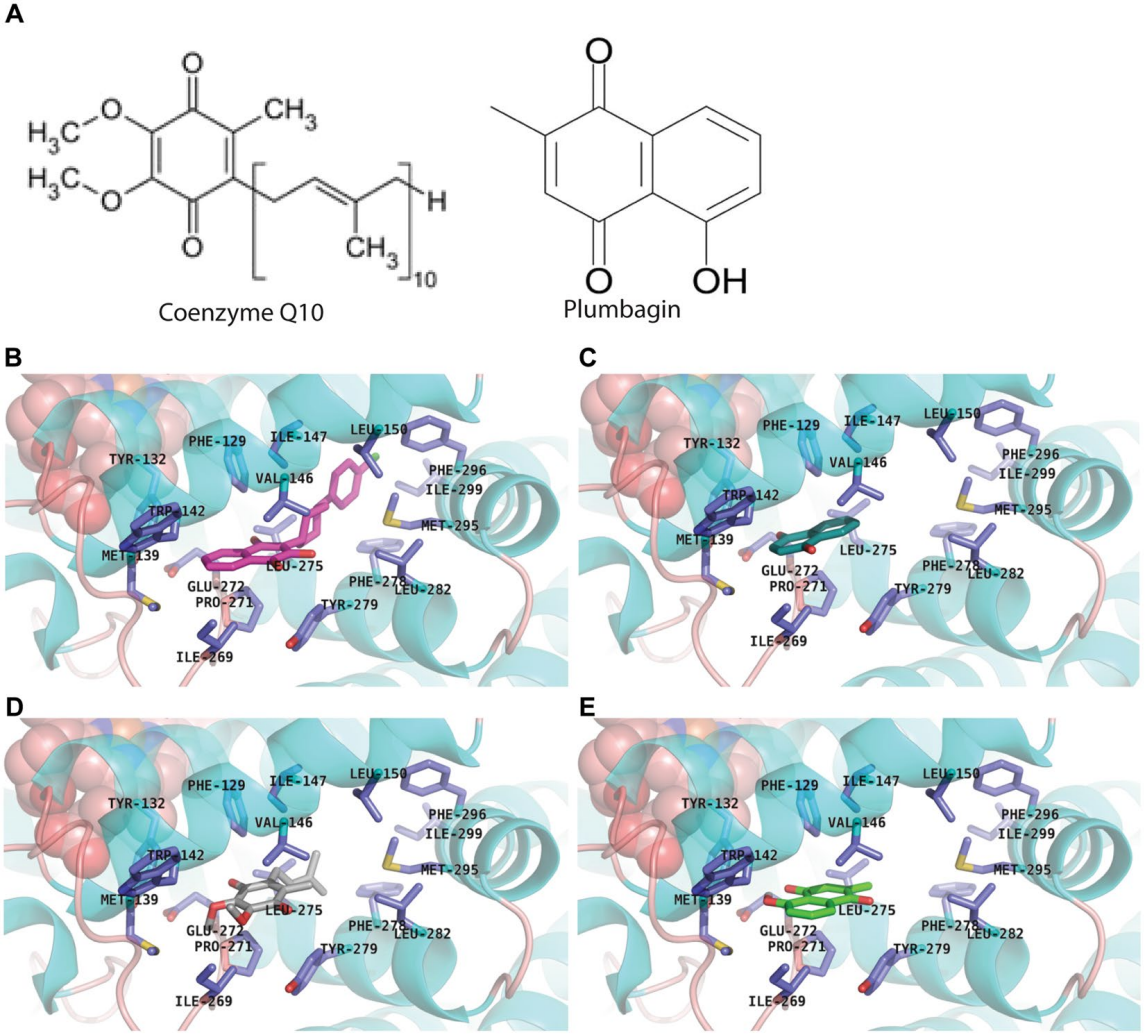
Benzoquinone/naphthoquinone substructure placement of docked ligand poses is consistent with co-crystallized ligand at the Q_0_ site of Complex III. (**A**) Chemical structures of plumbagin and CoQ. (**B**) Using the atovaquone-bound (magenta carbons) form of the Complex III x-ray crystal structure as a reference, we docked (**C**) 1,4-naphthoquinone (teal), (**D**) ubiquinone-1 (grey), and (**E**) plumbagin (green) for comparison of benzoquinone placements. Each docked compound produced a favorable pose with its benzoquinone/naphthoquinone moiety occupying the same region as atovaquone’s naphthoquinone substructure within the Q_0_ site. Sidechains in the binding site are shown as sticks with dark blue carbons.

Next, the mechanism through which plumbagin increases intracellular oxygen radicals was investigated. The mitochondria are the most prominent site for oxygen radicals in the cells (reviewed in refs^10,11^). Primarily, oxygen radicals are produced at the mitochondrial complexes I and III and as demonstrated in recent studies, also by complex II of the electron transport chain^12^. These complexes contain sites for the electron carrier, CoQ (ubiquinone). The benzoquinone portion of CoQ (Fig. 5A) is required for the shunting of electrons between the first three complexes of the electron transport chain. Since plumbagin is a naphthoquinone, we hypothesized that this compound might interfere with electron transport via mimicry of the CoQ benzoquinone headgroup.

To examine the plausibility of this molecular mechanism, we used molecular docking to examine plumbagin’s steric and electrostatic complementarity with the various CoQ binding sites (Q-sites) on Complexes I–III in the electron transport chain. Plumbagin binding poses were then inspected for qualitative agreement with experimentally observed benzoquinone-containing ligands at the Q-sites (from x-ray crystal structures) with respect to the benzoquinone positioning. Plumbagin was docked at the known Q-sites on mitochondrial Complexes II (site Q) and III (sites Q_0_ and Q_i_) and a putative Q-site on Complex I based on an extensive site-directed mutagenesis study looking at the complex function and inhibitor potencies^13^. In docking simulations, we observed favorable binding poses for plumbagin that were consistent with the positioning of the benzoquinone head groups of CoQ, naphthoquinone, and atovaquone (a complex III inhibitor^14,15^). For example, in the atovaquone-bound Q_0_-site of complex III (one of two different Q-sites in the complex), the docked poses of 1,4-naphthoquinone, ubiquinone-1 and plumbagin position their benzoquinone/naphthoquinone moieties in the same region as the naphthoquinone ring structure of atovaquone in the crystal structure (Fig. 5B–E). At an alternative Q_i_ site in complex III, we see the same consistent placement of benzoquinone moieties among docked analogues, in agreement with that of the ubiquinone-bound crystal structure (Supplementary File 4). A similar agreement was found in benzoquinone positioning for docked plumbagin and benzoquinone analogues with atpenin bound in its Complex II crystal structure at Q-site (Supplementary File 5). Finally, we considered a putative Q-site on Complex I near the interface of subunits PYYK and 49 kDa, as suggested previously^13^ and through our own inspection. Using the volume encompassing critical residues for inhibitor potencies as the docking search space, plumbagin docked favorably into a pocket near the Fe-S cluster N2 (Supplementary File 6). This pocket, adjacent to the inner mitochondrial membrane structure, is suitable for accommodating the benzoquinone moiety of the endogenous CoQ. These docking results support the plausibility of a competitive inhibitory mechanism for plumbagin at multiple Q-sites in the oxidative phosphorylation chain.

### Plumbagin decreases oxygen consumption rate

**Figure 6:**
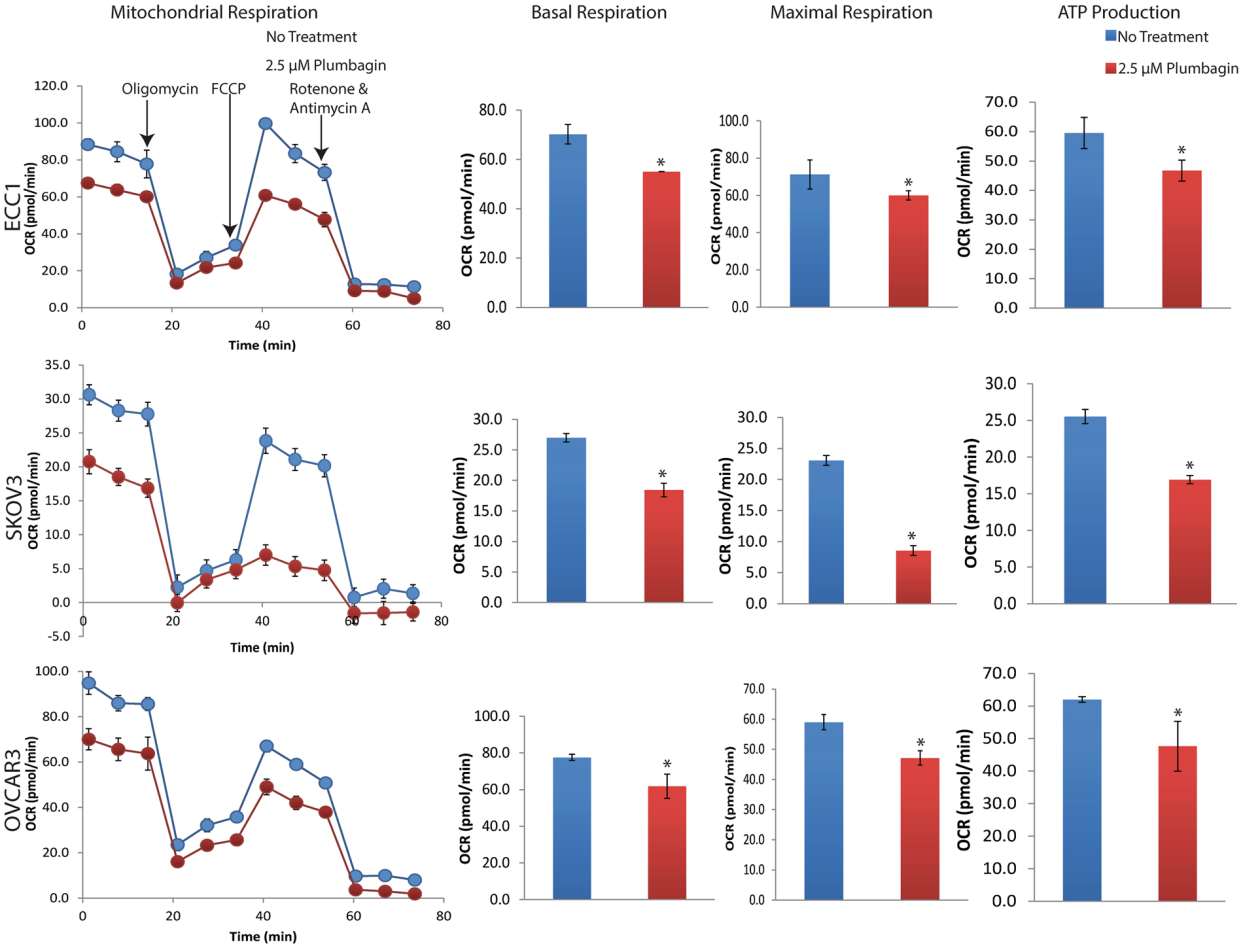
Plumbagin inhibits electron transport. ECC1, SKOV3 and OVCAR3 cells pre-incubated with plumbagin (2.5 μM) or vehicle control (DMSO) were monitored in Seahorse SF96 instrument for Oxygen Consumption Rate (OCR), basal and maximal respiration and ATP production. The concentrations of FCCP used for each cell line are as follows: ECC1 (0.25 µM), SKOV3 (0.10 µM), OVCAR3 (0.10 µM). Results shown are average from three biological replicates conducted with each cell line. p < 0.05. The oligomycin, FCCP, Rotenone and Antimycin treatment schedules used for all cells were identical to those used for ECC1.

To demonstrate plumbagin’s direct effect on electron transport, we monitored oxygen consumption rates in ECC1, SKOV3 and OVCAR3 cells treated with plumbagin. The cells were maintained in cell culture media or media containing plumbagin (2.5 μM) for 30 minutes prior to determining the oxygen consumption rate. At the outset of the experiment, we observed that the cancer cells exposed to plumbagin had approximately 25–35% lower basal oxygen consumption rate as compared to the media controls (Fig. 6). When ATP synthesis was uncoupled by promoting proton movement from the intermembrane space to the mitochondrial matrix using FCCP, the oxygen consumption rate in plumbagin-treated cells was increased. However, even under these conditions, the maximum oxygen consumption rate remained 25–40% lower than in the untreated cells (Fig. 6). Thus, the measurements clearly showed a decrease in oxygen consumption at both the basal level as well as at the maximum when proton transport was uncoupled (Fig. 6). In our hands, the cancer cells consistently exhibited decreased spare respiratory capacity as indicated by the peak oxygen consumption observed after the proton motive force was interrupted by FCCP. In addition to the decreased oxygen consumption rate, we also observed a decrease in ATP synthesis (Fig. 6). This observation suggests that plumbagin-mediated inhibition of the mitochondrial electron transport was decreasing ATP synthesis.

### Plumbagin decreases the optical redox ratio

**Figure 7:**
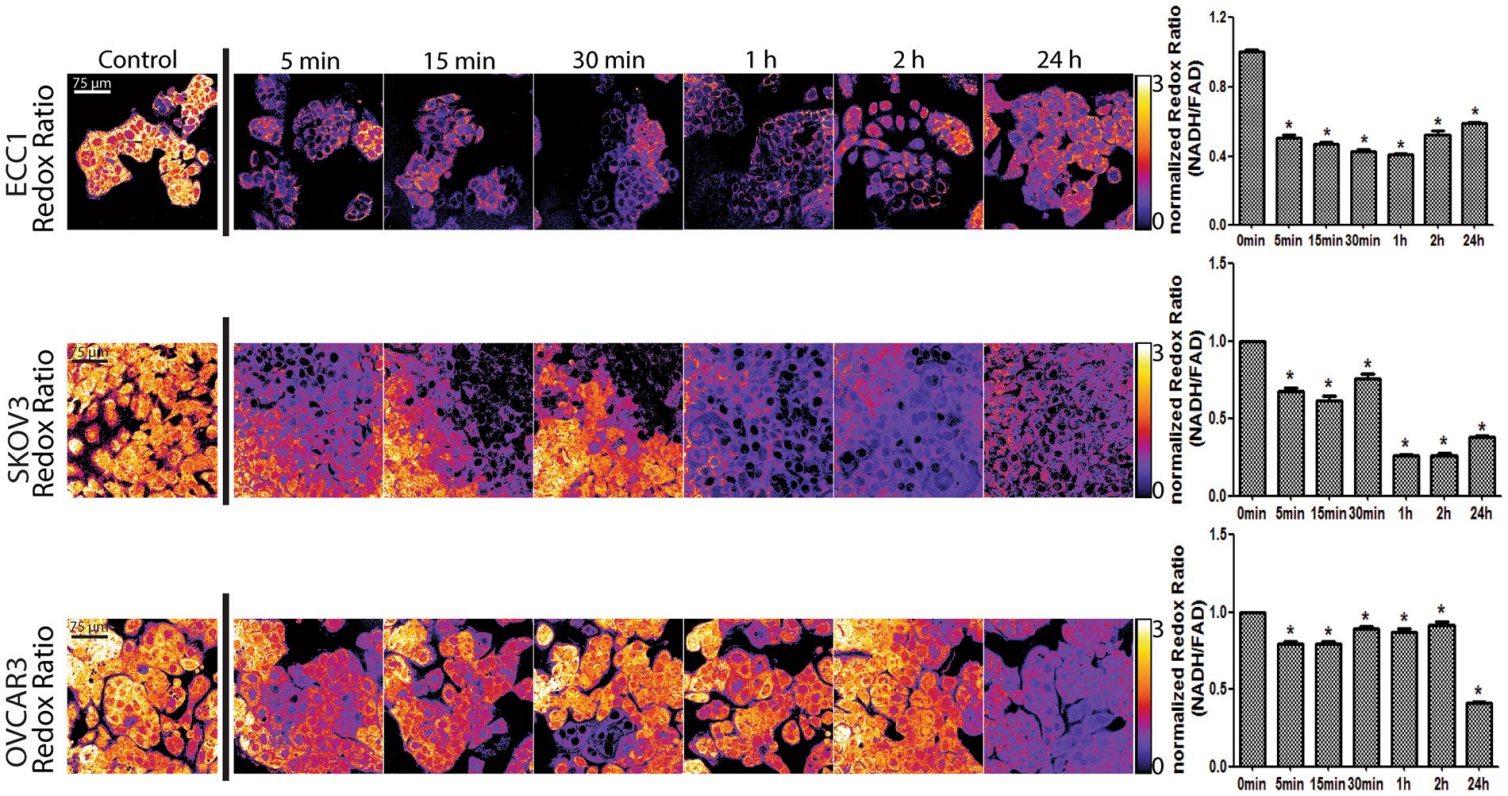
Plumbagin inhibits electron transport after complex II. Representative images of ECC1, SKOV3, and OVCAR3 cells show an immediate decrease in the optical redox ratio (NAD(P)H intensity/FAD intensity) post plumbagin treatment (5 μM). Quantitative analysis of the redox ratio confirms this decrease in optical redox ratio is due to a decrease in NAD(P)H intensity as shown in the bar graphs. Fluorescent images are representative images from three independent measurements. The data in the bar graphs is mean of all three independent experiments. The significance values (p-values) for the bar graphs are provided in the Supplementary File 7.

The optical redox ratio provides another measure of electron transport inhibition. ECC1 cells were treated with plumbagin and time course measurements were recorded to monitor NAD(P)H and FAD intensities. Representative redox images show a decrease in the optical redox ratio at all time points (Fig. 7; Supplementary File 7). Quantitative analysis of the optical redox ratio confirms significant decreases during the treatment time-course (p < 0.005, Fig. 7). The decrease in the redox ratio is due to a decrease in NAD(P)H intensity, and an increase in FAD intensity (Fig. 7). This observation is consistent with inhibition of electron transport after complex II^16^.

### Activation of Nrf2 is a protective response induced by the cancer cells to counter plumbagin-mediated oxidative stress

Time course studies showed that the level of oxygen radicals normalized to baseline levels by 2 h after treatment with plumbagin (data not shown). This time course was similar to that we have already demonstrated in our experiments with the monoterpene citral^17^. In the case of citral, we attributed the decrease in oxygen radicals to a compensatory increase in the intracellular levels of the anti-oxidant glutathione. Recent studies indicate that citral also induces an increase in the levels of the anti-oxidant, Nrf2 (Kapur *et al*., manuscript in preparation). With that knowledge, we tested if plumbagin could also induce a compensatory anti-oxidant response via Nrf2.

**Figure 8:**
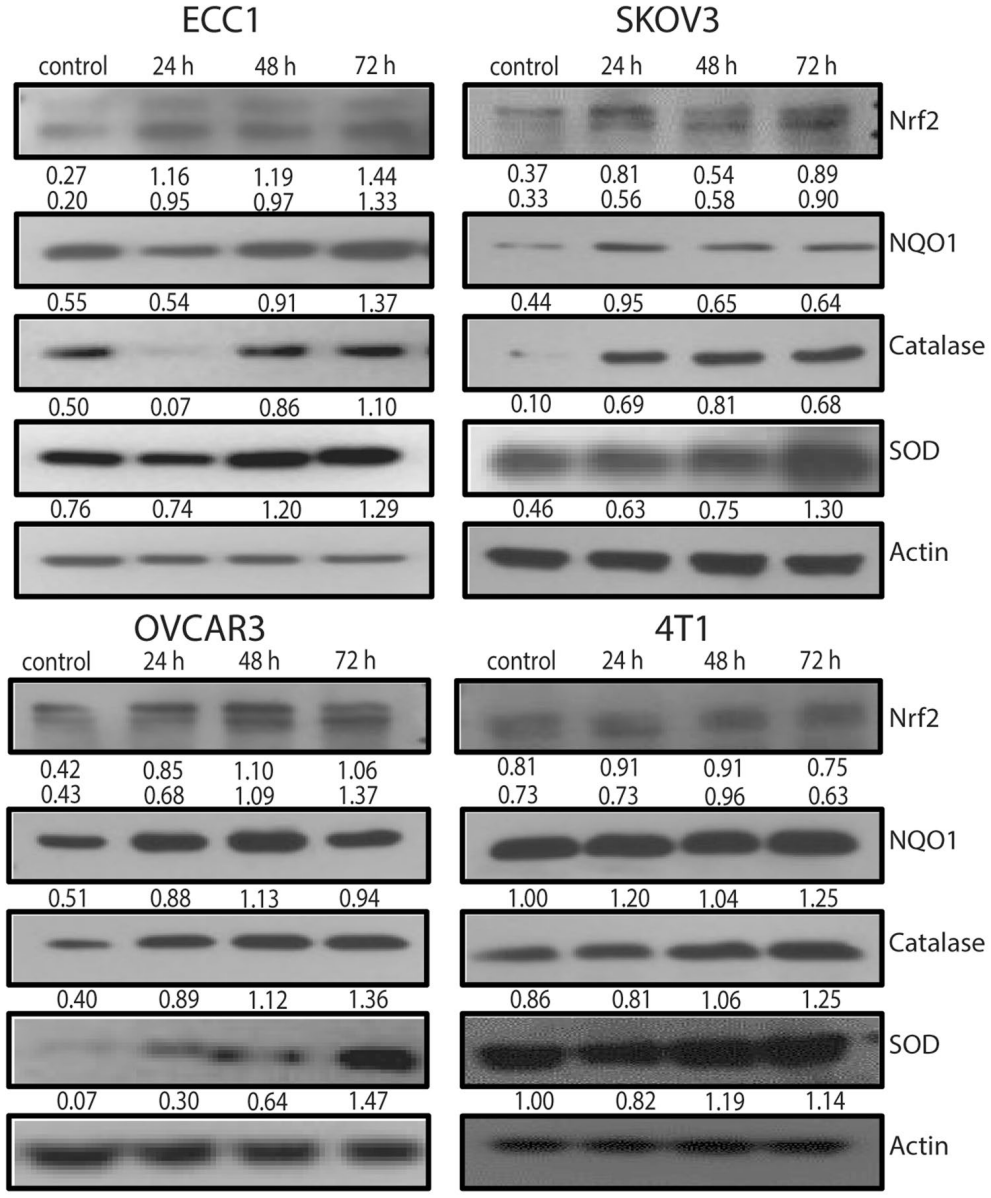
Inhibition of Nrf2 enhances plumbagin activity. ECC1, SKOV3, OVCAR3 and 4T1 cells treated with plumbagin (2.5 μM) show an increased expression of Nrf2 and its target genes NQO1, catalase, and SOD. Representative western blots from three biological replicates are shown. The full blots are presented in Supplementary File 10. (**B**) Numerical values under the blots are densitometry readings normalized to β-actin.

The transcription factor, Nrf2, is a master regulator of cellular anti-oxidant pathways including the synthesis of glutathione, catalase, superoxide dismutase and hemoxygenase-1. Western blots showed a significant and steady increase in Nrf2 levels in ECC1, SKOV3, OVCAR-3 and 4T1 cells exposed to plumbagin (Fig. 8). The Nrf2 regulated anti-oxidant genes NQO1, SOD and catalase were also increased at these time points indicating that the elevated Nrf2 levels correlated with an increase in the function of this transcription factor (Fig. 8). Although Nrf2 levels in 4T1 cells were not significantly increased (Fig. 8), there was a definite elevation of Nrf2-regulated genes (NQO1, catalase and SOD1) suggesting that even in this cell line, plumbagin treatment was triggering an anti-oxidative response.

### Inhibition of Nrf2 enhances plumbagin activity

Increased activity of Nrf2 and the anti-oxidant response proteins could dampen the effects of plumbagin on the cancer cells. We, therefore, tested if inhibition of Nrf2 activity would result in an increase in the cytotoxicity of plumbagin.

**Figure 9:**
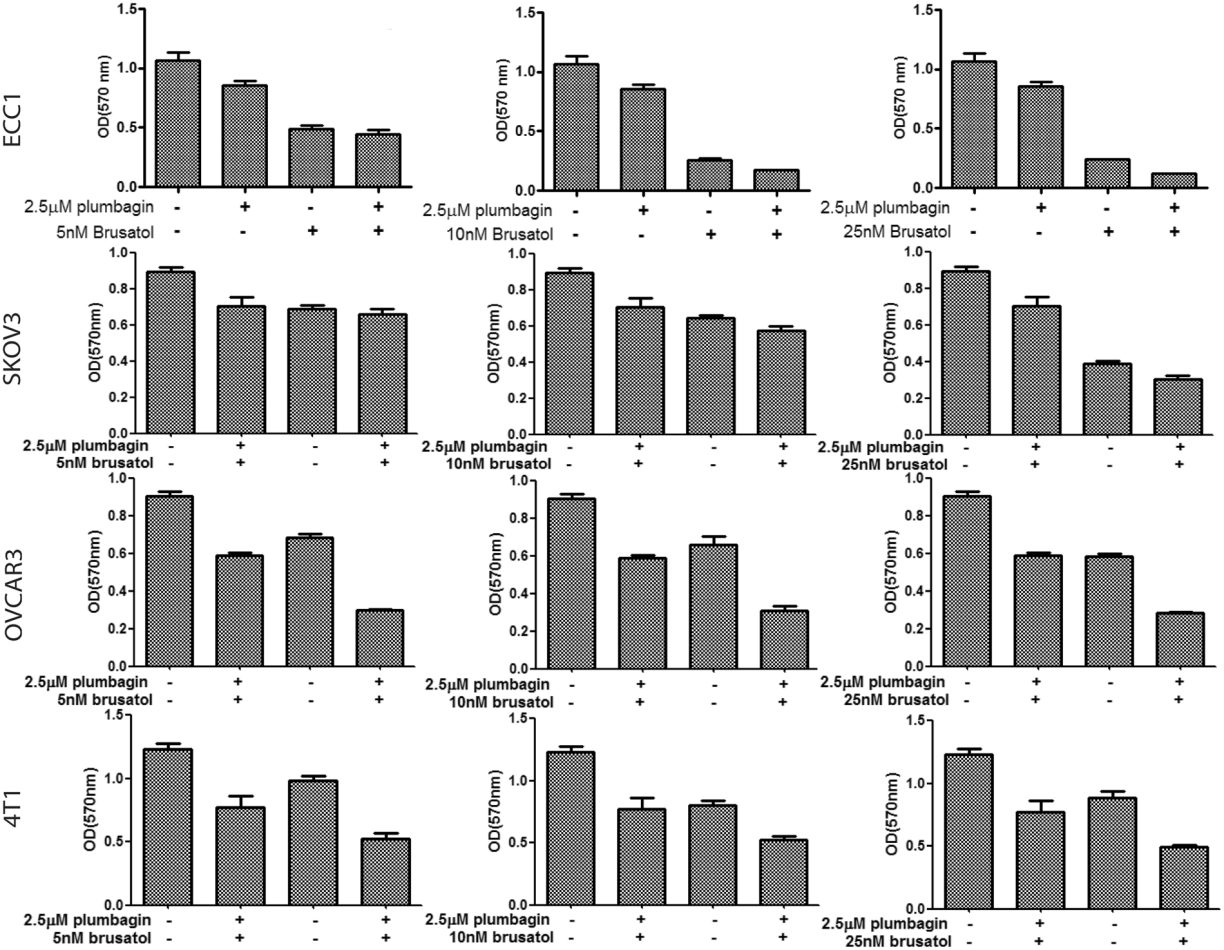
Enhanced decrease in viability following combined treatment with plumbagin and brusatol. MTT assays with ECC1, SKOV3, OVCAR3 and 4T1 cells were conducted to demonstrate increased effect on viability when cells were treated with a combination of plumbagin and brusatol. Assays were conducted over a 48 h time period. Each biological replicate contained eight technical replicates. Bar graphs show average of the three biological replicates for each cell line. P-values for all graphs are provided in Supplementary File 8.

Brusatol is a small molecule agent that specifically downregulates the expression of Nrf2^18,19^. We conducted MTT assays where ECC1, SKOV3, OVCAR3 and 4T1 cells were treated with a combination of plumbagin and brusatol (Fig. 9; Supplementary File 8). In all cell lines tested, combination of the two agents resulted in a significant increase in the inhibition of cell viability.

### Plumbagin synergizes with Nrf2 inhibitor, brusatol

**Figure 10:**
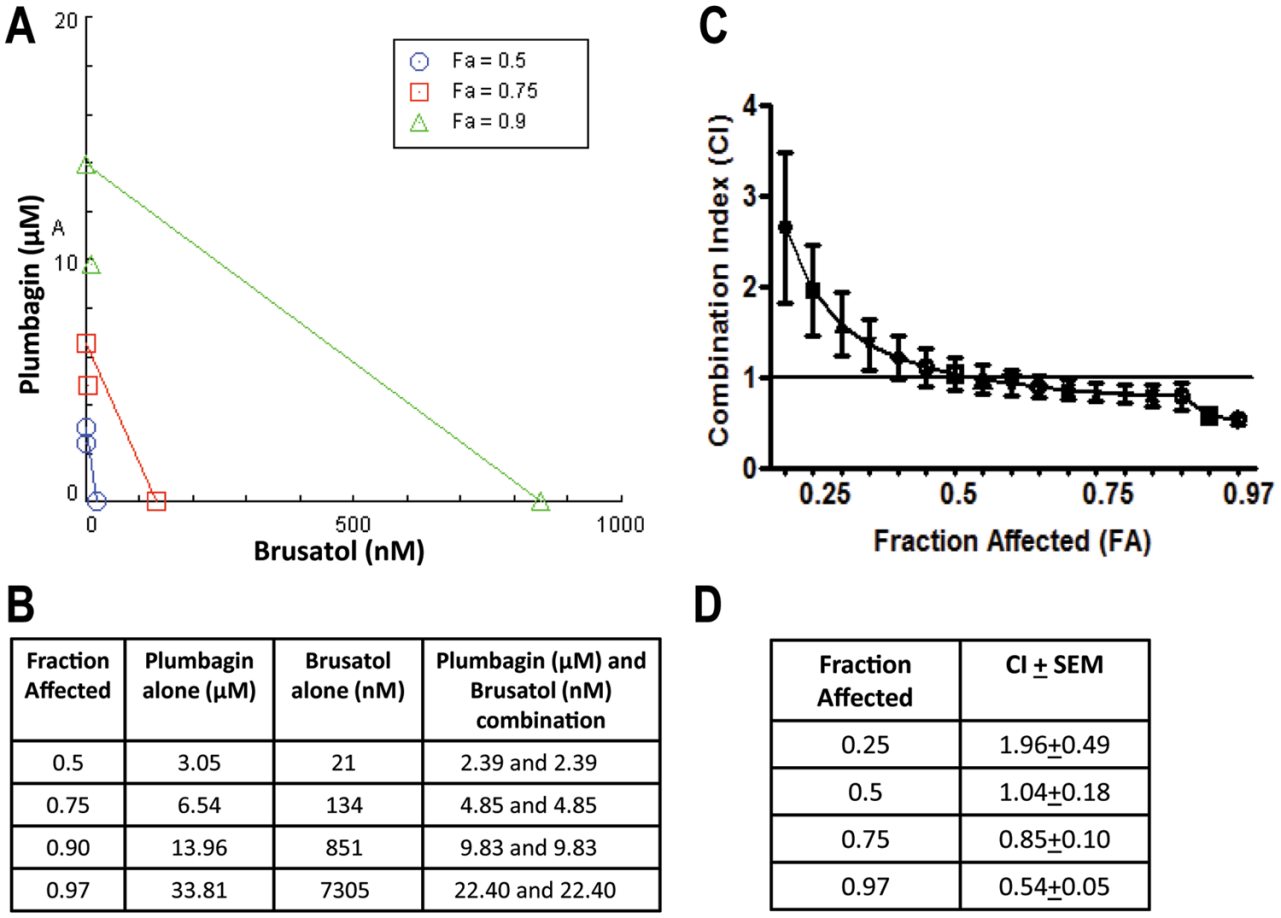
Brusatol synergizes with plumbagin to inhibit proliferation. The Chou-Talalay method was used to determine synergy between plumbagin and brusatol. MTT assays were conducted on ECC1 cells using varying concentrations of plumbagin and brusatol. Isobolograms (**A**) were constructed to determine concentration of the two drugs when used as single agents or in combination for the respective FAs (**B**). GraphPad Prizm was used to determine the CI values for the corresponding FAs (**C**,**D**).

Next, we used the Chou-Talalay algorithm^20–22^ to demonstrate synergism between plumbagin and brusatol using the MTT assays. Since all cell lines were showing increased inhibition of viability upon combined treatment with plumbagin and brusatol (Fig. 9), we employed ECC1 cells as a prototype to determine synergism between plumbagin and brusatol. A series of five different combinations of the two drugs were tested. Isobolograms constructed from this experiment demonstrated significant synergy between the two drugs when the viability of greater than 50% of the cells (Fraction affected, FA > 0.5) was adversely affected (Fig. 10A, B). The Combination Index for FA 0.75 and 0.97 was calculated to be 0.85 and 0.54 (Fig. 10C, D). Based on the parameters of the Chou-Talalay calculations, a CI of 1 indicates additive effects whereas CI < 1 shows synergy and CI > 1 is an indicator of antagonistic effects of the two drugs tested^21,22^. Since the CI for 0.75 and 0.97 FA was clearly < 1, there is significant synergy between plumbagin and brusatol. As shown in Fig. 10B, significantly less concentration of plumbagin and brusatol was required for FA 0.75, 0.9and 0.97 than when the two agents were used independently.

### Combined use of plumbagin and brusatol leads to increased oxidative stress

**Figure 11:**
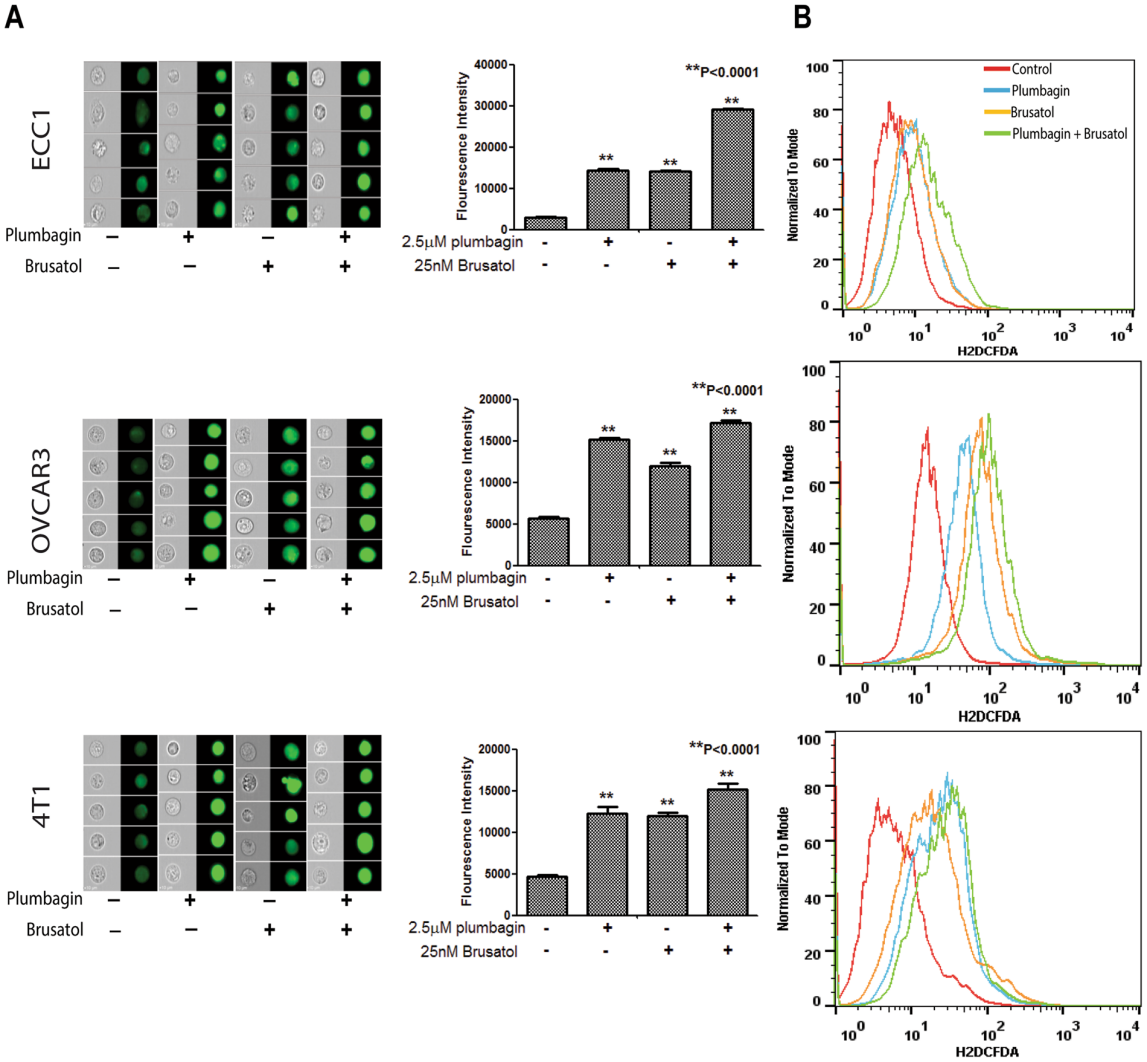
Inhibition of Nrf-2 increases plumbagin-induced oxidative stress. Combined treatment of ECC1, OVCAR3 and 4T1 cells with plumbagin and brusatol results in a sustained increase in intracellular oxygen radicals as determined by imaging cytometry (**A**) and flow cytometry (**B**). Data shown in all figures is representative of three biological replicates. Each biological replicate included three technical replicates.

**Figure 12:**
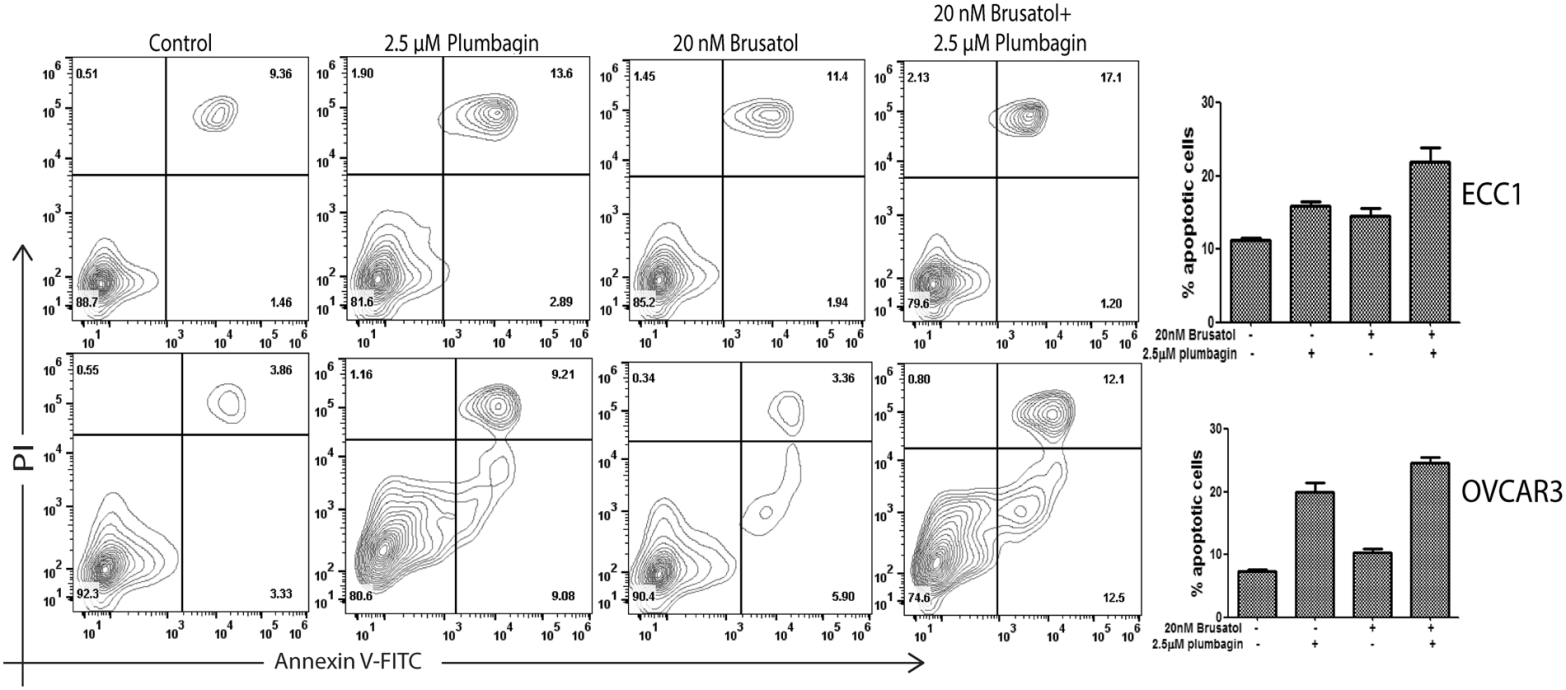
Combined treatment with plumbagin and brusatol increases apoptosis. Combined treatment of ECC1 and OVCAR3 cells with plumbagin and brusatol resulted in increased apoptosis as determined by flow cytometry. The contour plots are representative of three biological replicates. Each biological replicate contained three technical replicates. The bar graphs are average of the three biological replicate experiments. P-values for the bar graph are included in Supplementary File 9.

Next, we monitored intracellular oxygen radical levels in ECC1, OVCAR-3 and 4T1 cells treated with plumbagin and brusatol as single agents and in combination. Imaging cytometry demonstrated that combined treatment with plumbagin and brusatol significantly increased intracellular oxygen radicals in all three cell lines as compared to experiments where the cells were treated with plumbagin or brusatol alone (Fig. 11A). Flow cytometry experiments (Fig. 11B) also showed an increase in intracellular oxygen radicals in two (ECC1 and OVCAR3) of the three cell lines treated with the plumbagin plus brusatol combination. Finally, we also demonstrate that combined use of plumbagin and brusatol resulted in a 5–10% increase in apoptosis in ECC1 and OVCAR-3 cells as compared to experiments where these cell lines were treated with plumbagin alone (Fig. 12; Supplementary File 9).

## Discussion

Plumbagin inhibits *in vitro* proliferation and *in vivo* tumor growth of breast and prostate cancers, glioma, leukemia and other types of cancer^5,23–27^. In addition to its anti-cancer effects, plumbagin also causes apoptosis in malaria, leishmania, schistosomes and other protozoans^28,29^. In fact, plumbagin belongs to a class of phytochemicals that constitute a natural defense against parasites^30,31^. These previous reports suggest the possibility that plumbagin manifests its chemotoxic effects in cancer cells and parasites by targeting a central mechanism that is likely evolutionarily retained in eukaryotes.

An immediate effect of plumbagin in cancer cells is the significant spike in intracellular oxygen radicals. Inhibition of oxidative stress attenuates plumbagin activity in cancer cells, as shown in the current study (Figs 3 and 4) and by previous investigations with hepatocellular and lung cancer cells, as well as parasites^32,33^. Induction of oxidative stress in cells, therefore, appears to be an inherent property of plumbagin irrespective of targeted organism or cell type. However, the mechanism by which this molecule triggers an increase in oxygen radicals has not been investigated.

CoQ is required for electron transport from NADH and FADH_2_ between mitochondrial complexes I and III and II and III, respectively. Inefficient transfer of electrons at these complexes can lead to the formation of oxygen radicals. The quinone ring of CoQ is chemically similar to the 1,4-naphthoquinone unit of plumbagin (Fig. 5). Molecular docking experiments showed that plumbagin can dock to the CoQ binding sites (Q_0_ and Q_i_) (Fig. 5). In other words, plumbagin may interfere with CoQ-mediated electron transport.

Experimental proof of plumbagin’s effect on electron transport was confirmed by the reduced oxygen consumption and ATP synthesis (Fig. 6). Multiphoton autofluorescence microscopy further demonstrated a decrease in the redox ratio, corresponding to a decrease in NAD(P)H and increase in FAD signal intensity (Fig. 7). The decrease in NAD(P)H indicated that its oxidation to NAD(P) (the oxidized form not detected by multiphoton autofluorescence microscopy) by Complex I is not significantly inhibited by plumbagin. Similarly, the increase in FAD indicated that plumbagin was not interfering with the ability of Complex II to oxidize FADH_2_. The decrease in oxygen consumption and the block in electron transport is, therefore, likely to be due to inhibition of complex III by plumbagin. Additional biochemical experiments are currently underway to conclusively demonstrate that plumbagin is a complex III inhibitor. This observation explains the conservation of plumbagin activity in parasites and mammalian cells.

Previous studies have shown that plumbagin activates p53^34^. In our on-going experiments, we are testing if the activation of p53 is the direct result of DNA damage occurring when plumbagin increases the level of intracellular oxygen radicals in the cancer cells. Similarly, many of the previously studied effects of plumbagin are likely to be the result of oxidative stress. For example, plumbagin-mediated decrease in NF-κB expression and function^4,8,35^ may be ascribed to the ability of oxygen radicals to degrade IκBα and oxidize the Cys-62 residue of the p50 subunit to inhibit the transcriptional activity of NF-κB^36–39^. We, therefore, propose that oxidative stress is the central upstream event required for plumbagin’s chemotoxicity.

The high metabolic rate of cancer cells makes them particularly vulnerable to oxidative stress as compared to normal tissues^40^. Therefore, plumbagin and its analogs maybe effective as therapeutic agents that can target multiple tumor types. However, an oxidative environment decreases KEAP-1-facilitated proteasomal degradation of Nrf2 and increases nuclear translocation of this transcription factor^41,42^. Oxidative stress-induced increase in Nrf2 and its targeted genes, catalase and SOD detected in the current study (Fig. 8) is analogous to observations demonstrated previously in a cerebral ischemia model^43^. Therefore, activation of Nrf2 is a chemoresistance mechanism that compromises the anti-cancer activity of plumbagin. The combination of Nrf2 inhibitor brusatol and plumbagin produces a synergistic inhibition of cancer cell proliferation as demonstrated by the isobolograms in Fig. 10A. These data suggest that when plumbagin was used as a chemotherapeutic against tumors, its activity may be enhanced by co-administration of an Nrf2 inhibitor. On the other hand, when used as a chemopreventive agent (in subjects with high genetic risk for cancer, for example), the oxidative stress-induced anti-oxidative effect (triggered by increased Nrf2 activity) would be beneficial in avoiding DNA damage. Our study demonstrates this important distinction as plumbagin and its chemical analogs are considered in chemotherapeutic or chemopreventive settings. The targeting of electron transport may also explain the major toxicity associated with plumbagin. The understanding that plumbagin is targeting the mitochondrial electron transport complexes will allow designing of more potent plumbagin analogs that can be selectively targeted to the tumor cells and hence have a more acceptable toxicity profile.

## Methods

### Reagents and Cell Lines

DMEM (Dulbecco’s Modification of Eagle’s Medium), RPMI-1640, Hanks Balanced Salt Solution (HBSS), and Dulbecco’s Phosphate Buffered Saline (DPBS) from Cellgro (Manassas, VA) were the primary tissue culture media. SuperSignal West Dura Extended Duration Substrate was used for detection of protein bands on Western blots. RIPA buffer and Protease Inhibitor Cocktail were from ThermoFisher Scientific (Waltham, MA). All primary and secondary antibodies were from Cell Signaling Technology (Beverly, MA) or Jackson ImmunoResearch Laboratories (West Grove, PA), respectively. FITC-Annexin V Apoptosis Detection kit was purchased from BD Pharmingen (San Diego, CA). OVCAR3, ECC1, SKOV3, MCF7 and 4T1 cell lines were purchased from ATCC. The MYC-HRAS MOSE murine ovarian cancer cell line was a gift from Dr. Christine Walsh and was generated by introducing mutant c-myc and H-Ras genes into normal murine ovarian epithelial cells^44^.

### Cell Line Authentication

All previously established cell lines were purchased from ATCC (Manassas, VA) and maintained in the recommended culture media. Experiments with the majority of the cell lines were conducted within six months to one year of their purchase from ATCC. The cell lines not fitting this criterion were validated by Single Tandem Repeat (STR) analysis (Genetica DNA Laboratories, Burlington, NC). Cell line authentication by STR analysis was conducted not more than six months prior to their use in the reported experiments.

### Cell viability assays

Cell proliferation was monitored by the MTT assay as described previously^17,45–47^. Briefly, cells (2,500–5,000 cells/well) were treated with the test compounds for 24, 48 or 72 h followed by addition of MTT (Sigma, St. Louis, MO; 0.5 mg/ml final concentration) to each well. After incubation for 3 h, the media was removed, DMSO (100 μl) was added to each well and absorbance (570 nm) was determined on a Spectra MAX 190 (Molecular Devices, Sunnyvale, CA) plate reader.

### Annexin V assays

Apoptosis in cells treated with plumbagin was determined by flow cytometry as described^47^. Briefly, cells treated with plumbagin for 18 h were labeled with Annexin V-FITC and propidium iodide and analyzed by flow cytometry on a BD FACSCalibur instrument. Data were analyzed using FlowJo software (FlowJo LLC, Ashland OR). Percentage of early (Annexin V^+^/propidium iodide^−^) and late (Annexin V^+^/propidium iodide^+^) apoptotic cells in the control and treated groups were determined. All experiments were repeated three times.

### H2AX flow cytometry

Cells (2 × 10^6^) were plated in 10 cm plates and treated with 2.5 μM plumbagin for 24 hrs. Post treatment cells were harvested using trypsin and stained intracellularly for phosphoH2AX (Antibody obtained from Cell Signaling) using standard method of staining. The data was acquired using Attune Flow cytometer and analyzed using FlowJo software.

### DNA damage Gel electrophoresis

Cells from the previous experiment were divided into two, one sample was used to stain for H2AX and the other sample was used to isolate the DNA using quick DNA extract reagent from Epicenter. The DNA was quantified using the Nanodrop 2000 and equal amount of DNA was loaded on 0.8% Agarose gel and run for one hour at 70 V. The bands were visualized using FluorChem 8900 Imaging System (Alpha Innotech, Santa Clara, CA) and the images were processed using the ImageJ software.

### Western blotting

Cells (5 × 10^5^) were treated with plumbagin, washed with ice-cold PBS and lysed in RIPA buffer containing protease inhibitors. Protein concentration was measured using BCA protein assay (ThermoFisher, MA). Lysates equivalent to 25 μg of total protein were electrophoresed and blotted onto PVDF membranes that were probed with primary and secondary antibodies. The proteins were detected by autoradiography using chemiluminescence substrate. The autoradiography films were scanned using FluorChem 8900 Imaging System (Alpha Innotech, Santa Clara, CA) and the images were processed using the ImageJ software.

### Monitoring intracellular oxygen radicals

Increase in the level of intracellular oxygen radicals in response to plumbagin was determined by flow and imaging cytometry as described^17,47^. ECC1 cells were incubated with 10 μM H_2_-DCFDA (Molecular Probes, OR) for 30 min at 37 °C, followed by treatment with plumbagin. The cells were washed with PBS, harvested and ROS content was measured as the fluorescent DCF product on Imagestream II (EMD Millipore, Darmstadt, Germany) imaging cytometer or FACSCalibur (BD Biosciences, CA) flow cytometer. For Imagestream, data were acquired using INSPIRE V.200.1.388.0 acquisition software, using Channel1 for Bright Field and Channel 2 (533/55 filter) for green fluorescence. The images were acquired at a magnification of 40× with 488 illumination at 5 mW and 785 nm illumination at 1.72 mW laser power. Results from imaging cytometry were analyzed by using the proprietary IDEAS V 6.1.303.0 analysis software package and FACSCalibur flow cytometry data were analyzed using FlowJo software (Ashland, OR).

### Molecular docking of plumbagin with mitochondrial complexes

Complex structures were downloaded from RCSB PDB website: Complex I (unliganded mitochondrial complex I (sheep), PDB accession code 5LNK), Complex II (atpenin-bound mitochondrial complex II (wild boar), PDB accession code 3AEE), Complex III (atovaquone- and ubiquinone-6-bound cytochrome bc1 complex (mouse), PDB accession code 4PD4). Receptor structures were assigned polar hydrogens and partial charges using the prepare_receptor.py script provided with AutoDockTools v1.5.6^48^. Compound structures, plumbagin [PubChem CID10205], ubiquinone-1 [CID4462], 1,4 naphthoquinone [CID8530] were downloaded from PubChem (3D SD format) and assigned partial charges using OpenEye’s molcharge utility (MOL2 format) with the MMFF method (OpenEye Scientific Software, QUACPAC v1.6.3.1). The program Smina, a fork of AutoDock Vina v1.1.2, was used for ligand docking using the default search parameters and scoring function^49,50^. To designate a search space to explore around each possible ubiquinone reduction site (Q-site), a Q-site at Complex II (atpenin-occupied) and the Q_0_ and Q_i_ sites of Complex III (atovaquone and CoQ occupied, respectively), the *autobox ligand* feature was applied. This function automatically defines a search volume based on a specified co-crystallized ligand’s coordinates. To each of these sites, the co-crystallized ligand was re-docked to validate the docking procedure. In each case, the ligand’s crystallographic binding pose was reproduced to high precision (<1.0 Å RMSD). In Complex I, the binding site for CoQ has not been definitely established with a co-crystallized ligand. Therefore, a search volume encompassing a putative Q-site, based on extensive site-directed mutagenesis data^13^, was applied: centered at Cartesian position (78.6, 91.8, 197.0) with box edges of 28.0 Å. Output poses were inspected and rendered using PyMOL (The PyMOL Molecular Graphics System, Version 1.7.6.0 Schrödinger, LLC.).

### Multiphoton autofluorescence microscopy of the optical redox ratio

Fluorescence lifetime images were taken on a custom-built inverted multiphoton microscope (Bruker), as described previously^51,52^. Although fluorescence lifetimes were measured, only fluorescence intensity values are reported here for brevity. A titanium:sapphire laser (Coherent Inc.) was used for excitation, tuned to 750 nm to excite NAD(P)H and 890 nm to excite FAD. Note that the fluorescence intensities and lifetimes of NADH and NADPH overlap, and thus we refer to the fluorescence signal as NAD(P)H. However, most of the collected signal is due to NADH^53^. Both the excitation and emission light were coupled through a 40× water immersion (1.15NA) objective of the inverted microscope (Nikon, TiE). Customized bandpass filters were used for both NAD(P)H and FAD. The NAD(P)H bandpass filter isolated emission between 400 and 480 nm, while the FAD filter isolated between 500 and 600 nm. Fluorescence lifetime images were collected using time correlated single photon counting electronics (SPC-150, Becker and Hickl) and a GaAsP photomultiplier tube (H7422P-40, Hamamatsu). A pixel dwell time of 4.8μs was used to acquire a 256 × 256 pixel images over 60 s. The photon count rates were maintained above 5 × 10^5^ to ensure adequate photon observations for lifetime decay fits, and to ensure that no photobleaching occurred. Images were taken sequentially, first a NAD(P)H image and then an FAD image of the same field of view. A Fluoresbrite YG microsphere (Polysciences Inc.) was imaged daily as a standard for quality control, and the fluorescence lifetime of the bead, 2.11 ± 0.05 ns, agrees with published values^51,53^.

For the imaging experiments, cells were plated in RPMI (with 10%FBS and 1% Pen/Strep) on a 35 mm glass-bottom petri dish (MatTek Corp). Cells were then treated and imaged 24 h after plating. NAD(P)H and FAD fluorescent lifetime images of the cells were acquired directly through the bottom of the Petri dishes at an initial time point as the control. The media was then replaced with media containing 5 μM of Plumbagin (made fresh before imaging) and the cells were imaged 5 min, 15 min, 30 min, 1 h, 2 h, and 24 h post treatment.

A fluorescence intensity image was generated from the lifetime images by summing the photon counts per pixel over the 60 second collection time. The cytoplasm of the cells were segmented from the nuclei and background using an automated image segmentation algorithm^54^. The fluorescence intensity and optical redox ratio (NAD(P)H intensity/FAD intensity), were calculated for each cytoplasm.

### Assessment of the effect of plumbagin on mitochondrial respiration

SKOV3 (15,000/well) were seeded in a Seahorse XF-96 Analyzer (Agilent Technology, Santa Clara, CA) culture plate and after 24 h treated with 2.5 μM plumbagin for 30 min. Cells were washed and growth media was replaced with 175 µL of FX media (FX Assay Modified DMEM from Agilent Technologies with 5.5 mM glucose, 1 mM pyruvate, and 2 mM glutamine). After media exchange was complete, cells were equilibrated for 1 h at 37 °C in a non-CO_2_ incubator. The mitochondrial stress assay was performed with sequential injections of oligomycin A (1 μM, ATP synthase inhibitor); FCCP (0.07 and 0.10 μM, to uncouple proton gradient); and a mixture of 1 μM rotenone and 1 μM antimycin A (inhibitors of complex I and III, respectively). Oxygen consumption rates (OCR) and ATP production were measured with each drug injection series.

### Drug synergy assays

Cell viability (MTT) assays were used to determine synergy between plumbagin and brusatol. The IC_50_ of each drug was calculated against ECC1 cells using GraphPad Prizm software. These IC_50_ values were then used to set up combination concentrations for the two agents as indicated by the CompuSyn software. Plumbagin and brusatol concentrations tested for the synergy assays were as follows: plumbagin/brusatol: 8 µM/40 nM, 4 µM/20 nM, 2 µM/10 nM, 1 µM/5 nM, 0.5 µm/2.5 nM. MTT assays were conducted after 48 h treatment with the combination of plumbagin and brusatol. Combination Index (CI) and isobolograms were constructed using GraphPad Prizm and CompuSyn, respectively.

## Electronic supplementary material

Supporting data
