# Supplementary material for: Oxidative stress via inhibition of the mitochondrial electron transport and Nrf-2-mediated anti-oxidative response regulate the cytotoxic activity of plumbagin
Source: Sci Rep. 2018 Jan 18;8:1073. doi: 10.1038/s41598-018-19261-w (PMC5773707; doi:10.1038/s41598-018-19261-w)

Oxidative stress via inhibition of the mitochondrial electron transport and Nrf-2-mediated anti-oxidative response regulate the cytotoxic activity of plumbagin

Arvinder Kapur[1,*], Thomas Beres[1], Kavya Rathi[1], Amruta P. Nayak[1,2], Austin Czarnecki[1], Mildred Felder[1], Amani Gillette[3], Spencer S. Ericksen[4], Emmanuel Sampene[5], Melissa C. Skala[3], Lisa Barroilhet[1], and Manish S. Patankar[1,*]

Supplementary File 1

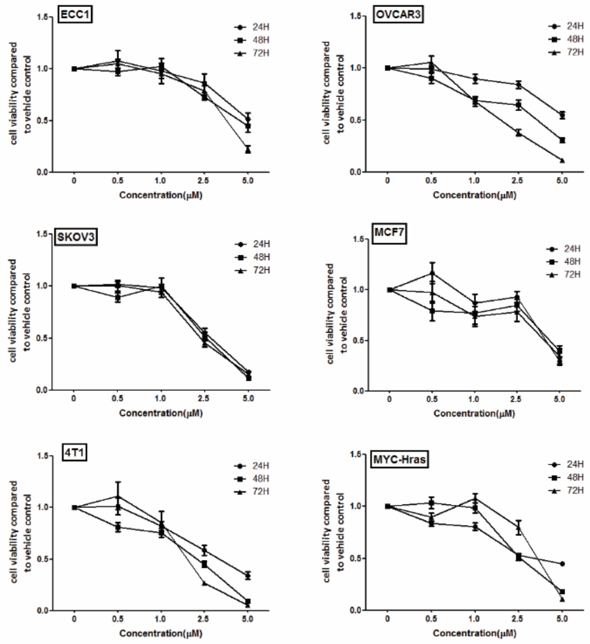

Statistical Analysis for MTT Assay in Different Cell Lines

		**0.5μM plumbagin**	**1.0 μM plumbagin**	**2.5μM plumbagin**	**5μM plumbagin**
	**24H**	NS	NS	NS	p<0.0001
**ECC1**	**48H**	NS	NS	p<0.0001	p<0.0001
	**72H**	NS	NS	p<0.0004	p<0.0001
		**0.5μM plumbagin**	**1.0 μM plumbagin**	**2.5μM plumbagin**	**5μM plumbagin**
	**24H**	NS	NS	NS	p<0.0001
**OVCAR3**	**48H**	NS	NS	p<0.0001	p<0.0001
	**72H**	NS	p<0.0001	p<0.0001	p<0.0001
		**0.5μM plumbagin**	**1.0 μM plumbagin**	**2.5μM plumbagin**	**5μM plumbagin**
	**24H**	NS	NS	p<0.0001	p<0.0001
**SKOV3**	**48H**	NS	NS	p<0.0001	p<0.0001
	**72H**	NS	NS	p<0.0004	p<0.0001
		**0.5 μM plumbagin**	**1.0 μM plumbagin**	**2.5μM plumbagin**	**5μM plumbagin**
	**24H**	NS	NS	NS	p<0.0001
**MCF7**	**48H**	NS	NS	NS	p<0.0001
	**72H**	NS	NS	NS	p<0.0002
		**0.5μM plumbagin**	**1.0 μM plumbagin**	**2.5μM plumbagin**	**5μM plumbagin**
	**24H**	NS	NS	P<0.0005	p<0.0001
**4T1**	**48H**	P<0.007	P<0.004	p<0.0001	p<0.0001
	**72H**	NS	NS	p<0.0002	p<0.0001
		**0.5μM plumbagin**	**1.0 μM plumbagin**	**2.5μM plumbagin**	**5μM plumbagin**
	**24H**	p<0.0001	p<0.0001	p<0.0001	p<0.0001
**MYC-HRAS**	**48H**	NS	NS	p<0.0001	p<0.0001
	**72H**	NS	NS	NS	p<0.0001

Supplementary File 2

CELL LINE	IC_{50} FOR PLUMBAGIN (μM)
ECC1	3.5
SKOV3	2.25
OVCAR3	1.5
MCF7	3.0
4T1	1.5
MYC-HRAS	3.5

Supplementary File 3

Significance values for Experiment to show inhibition of Plumbagin-induced Apoptosis by NAC (Figure 4)

	ECC1	SKOV3	OVCAR3	4T1
**control vs 2.5 μM plumbagin**	**P<0.01**	**P<0.03**	**P<0.0001**	**P<0.001**
**control vs 1 mM NAC**	**NS**	**NS**	**P<0.0001**	**P<0.01**
**control vs 1 mM NAC & 2.5 μM plumbagin**	**NS**	**P<0.03**	**P<0.009**	**NS**
**2.5 μM plumbagin vs 1 mM NAC**	**P<0.01**	**P<0.03**	**P<0.003**	**P<0.0001**
**2.5 μM plumbagin vs 1mM NAC & 2.5 μM plumbagin**	**P<0.01**	**P<0.05**	**P<0.003**	**P<0.0002**
**1 mM NAC vs 1 mM NAC & 2.5 μM Plumbagin**	**NS**	**NS**	**NS**	**P<0.04**

Supplementary File 4

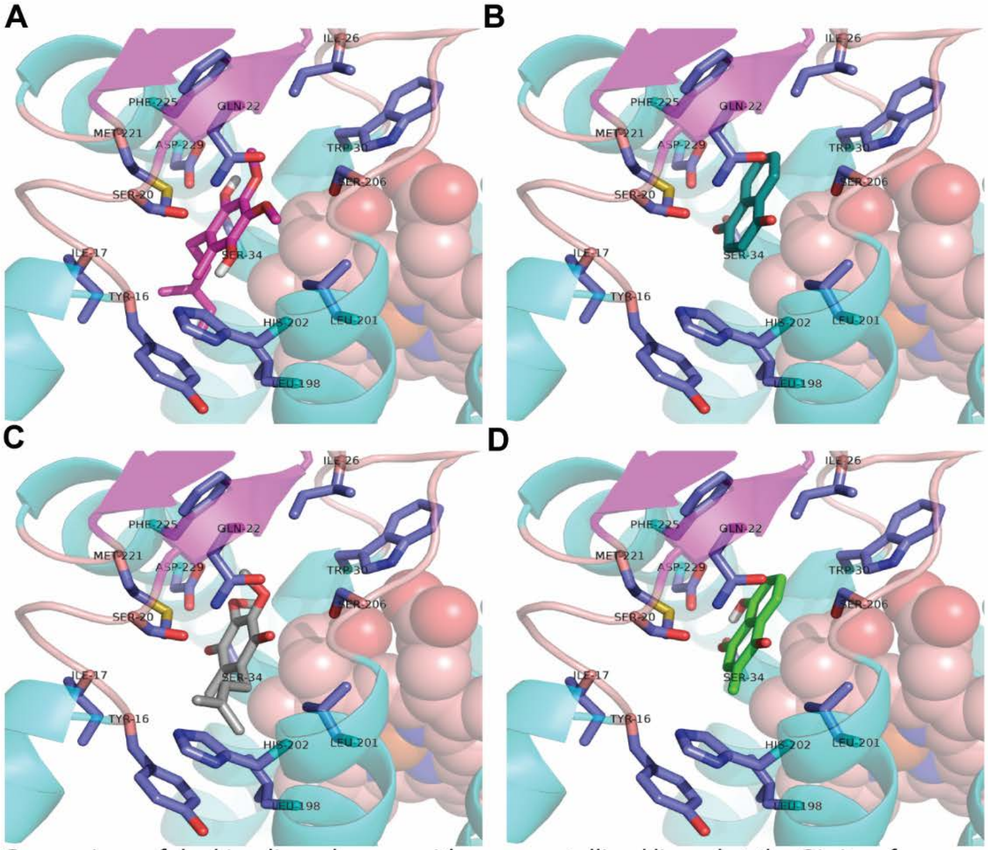

Comparison of docking ligand poses with a co-crystallized ligand at the Qi site of Complex III. (A) Using the ubiquinone-bound (magenta carbons) Complex III x-ray crystal structure as a reference, we docked (B) 1,4-naphthoquinone (teal), (C) ubiquinone-1 (grey), and (D) plumbagin (green). Each docked compound produced a favorable pose with its benzoquinone/naphthoquinone moiety occupying the same region as ubiquinone's benzoquinone substructure. Sidechains in the binding site are shown as sticks with dark blue carbons.

Supplementary File 5

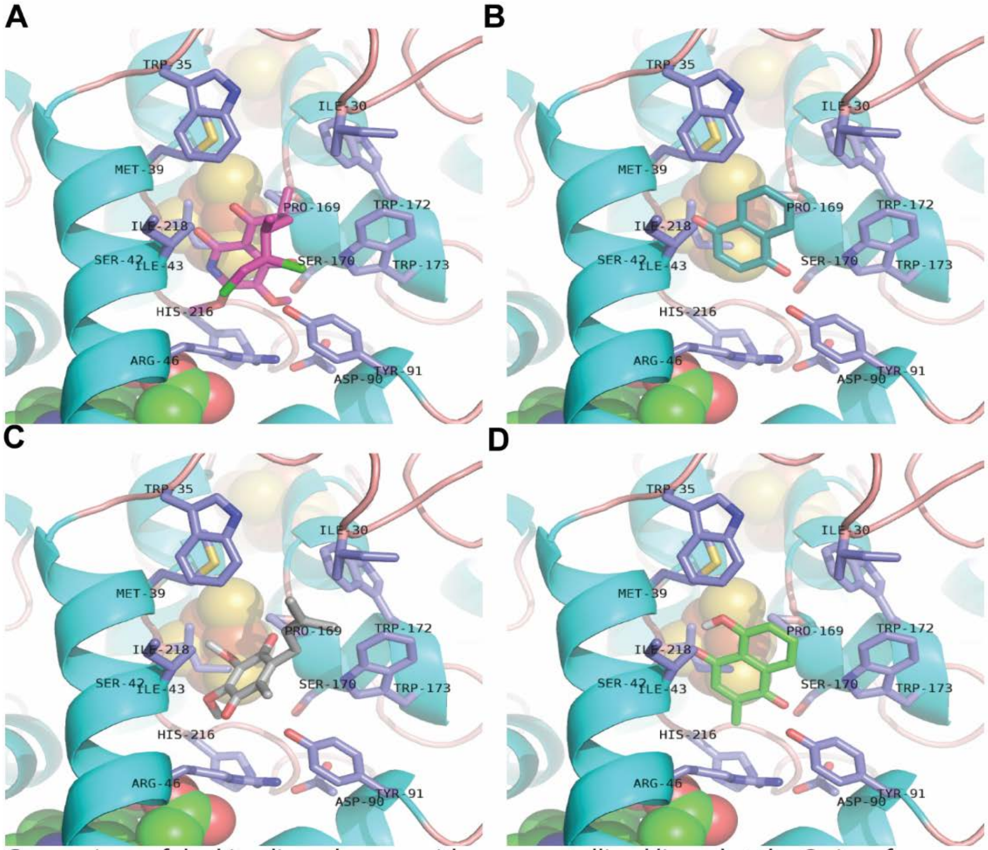

Comparison of docking ligand poses with a co-crystallized ligand at the Q site of Complex II. (A) Using the atpenin A5-bound (magenta carbons) Complex III x-ray crystal structure as a reference, we docked (B) 1,4-naphthoquinone (teal), (C) ubiquinone-1 (grey), and (D) plumbagin (green). Each docked compound produced a favorable pose with its benzoquinone/naphthoquinone moiety occupying a similar region as atpenin A5's pyridinone substructure. Sidechains in the binding site are shown as sticks with dark blue carbons.

Supplementary File 6

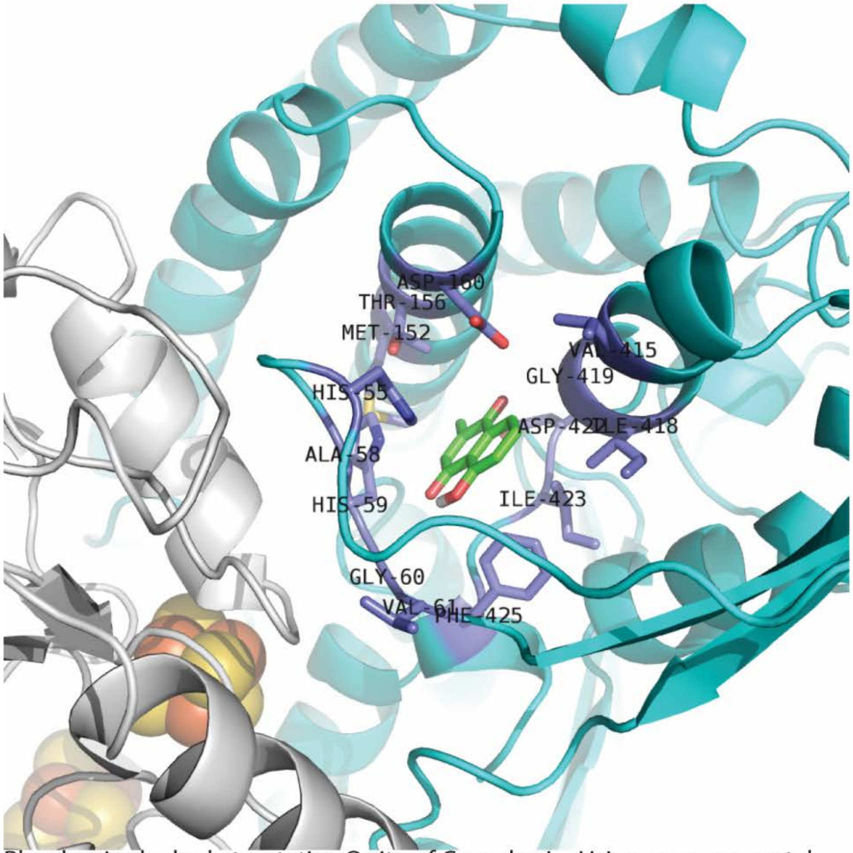

Plumbagin docked at putative Q site of Complex I. Using an x-ray crystal structure of a complete ligand-free complex I, a potential binding site for the benzoquinone portion of Coenzyme Q was identified near the interface of the subunits PYYK (white carbon) and 49 kDa (cyan carbon), as suggested by findings in Fendel et al, 2008 and our own inspection. Using the volume encompassing critical residues for inhibitor potencies as the docking search space, plumbagin (sticks, green carbons) docked favorably into a pocket near the Fe-S cluster N2 (yellow/orange spheres in lower left corner). Sidechains in the binding site are shown as sticks with dark blue carbons.

Supplementary File 7

Significance values for the Redox Ratios in the Multiphoton Autofluorescence Assay (Figure 7)

ECC1

P-Values	Redox Ratio
C-5m	3.09E-58
C-15m	2.27E-53
C-30m	1.04E-69
C-1h	3.30E-80
C-2h	1.40E-44
C-24h	2.94E-57

SKOV3

P-Values	Redox Ratio
C-5m	8.33933E-28
C-15m	5.00325E-20
C-30m	9.56113E-13
C-1h	3.0272E-148
C-2h	1.7804E-140
C-24h	1.0726E-154

OVCAR3

P-Values	Redox Ratio
C-5m	1.02422E-26
C-15m	1.51818E-22
C-30m	6.18262E-12
C-1h	7.5939E-11
C-2h	7.21231E-08
C-24h	9.5575E-173

Supplementary File 8

Significance values for MTT assay on different cells treated with combination of Plumbagin and Brusatol (Figure 9)

	ECC1	SKOV3	OVCAR3	4T1
control vs 2.5 μM plumbagin	P<0.01	P<0.005	P<0.0001	P<0.001
control vs 5 nM Brusatol	P<0.0001	P<0.0001	P<0.0001	P<0.001
2.5 μM plumbagin vs 5 nM Brusatol	P<0.0001	NS	P<0.002	P<0.06
2.5 μM plumbagin vs 2.5 μM plumbagin & 5nM Brusatol	P<0.0001	NS	P<0.0001	P<0.05
5 nM Brusatol vs 2.5 μM plumbagin & 5 nM Brusatol	P<0.0001	NS	P<0.0001	P<0.0001
control vs 2.5 μM plumbagin	P<0.01	P<0.005	P<0.0001	P<0.001
control vs 10 nM Brusatol	P<0.0001	P<0.0001	P<0.0003	P<0.001
2.5 μM plumbagin vs 10 nM Brusatol	P<0.001	NS	NS	NS
2.5 μM plumbagin vs 2.5 μM plumbagin & 10nM Brusatol	P<0.0001	P<0.0001	P<0.0001	P<0.03
10 nM Brusatol vs 2.5 μM plumbagin & 10 nM Brusatol	NS	P<0.0001	P<0.0001	P<0.0002
control vs 2.5 μM plumbagin	P<0.01	P<0.005	P<0.0001	P<0.001
control vs 25 nM Brusatol	P<0.0001	P<0.0001	P<0.0001	P<0.0009
2.5 μM plumbagin vs 25 nM Brusatol	P<0.0001	P<0.0001	NS	NS
2.5 μM plumbagin vs 2.5 μM plumbagin & 25 nM Brusatol	P<0.0001	P<0.0001	P<0.0001	P<0.01
25 nM Brusatol vs 2.5 μM plumbagin & 25 nM Brusatol	P<0.0001	P<0.002	P<0.0001	P<0.0001

Supplementary File 9

P-values for Apoptosis assay on ECC1 and OVCAR3 treated with combination of Plumbagin and Brusatol (Figure 12)

	ECC1	OVCAR3
**Control vs 2.5 μM plumbagin**	**p<0.002**	**P<0.0009**
**control vs 25nM Brusatol**	**p<0.02**	**P<0.01**
**2.5 μM plumbagin vs 25nM Brusatol**	**P<0.32**	**P<0.003**
**2.5 μM plumbagin vs 2.5 μM plumbagin &25nM Brusatol**	**P<0.04**	**P<0.05**
**25nM Brusatol vs 2.5 μM plumbagin &25nM Brusatol**	**P<0.02**	**P<0.0002**

Supplementary File 10 – Full blots for Figure 1(C)

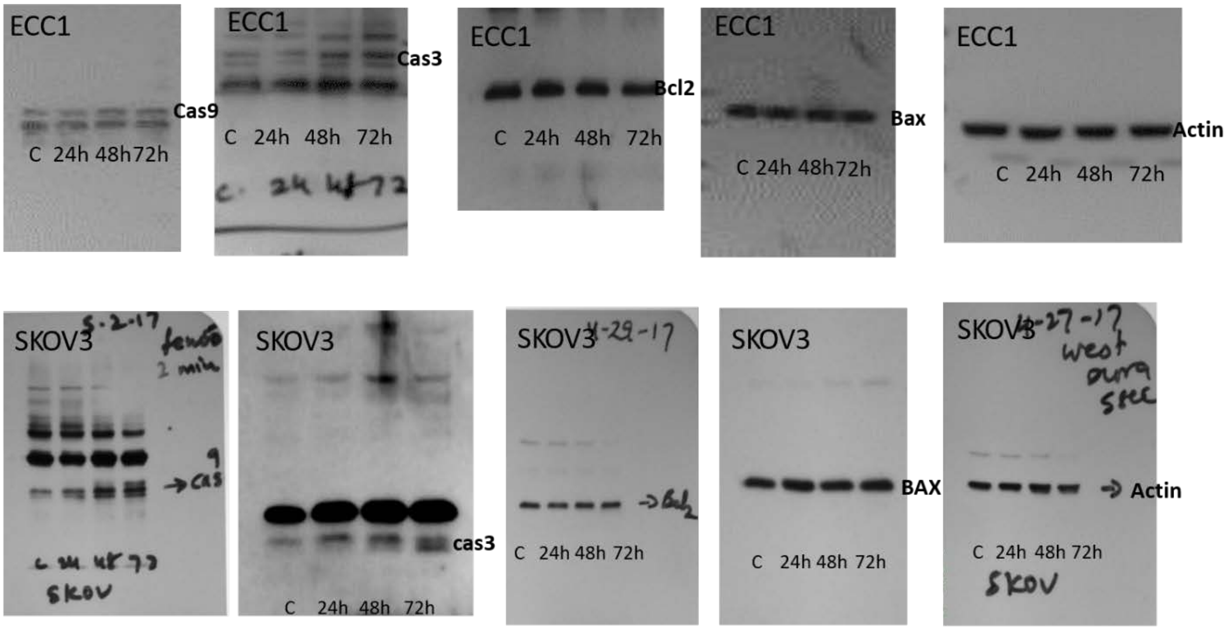

Supplementary File 10 – Full blots for Figure 1(C)

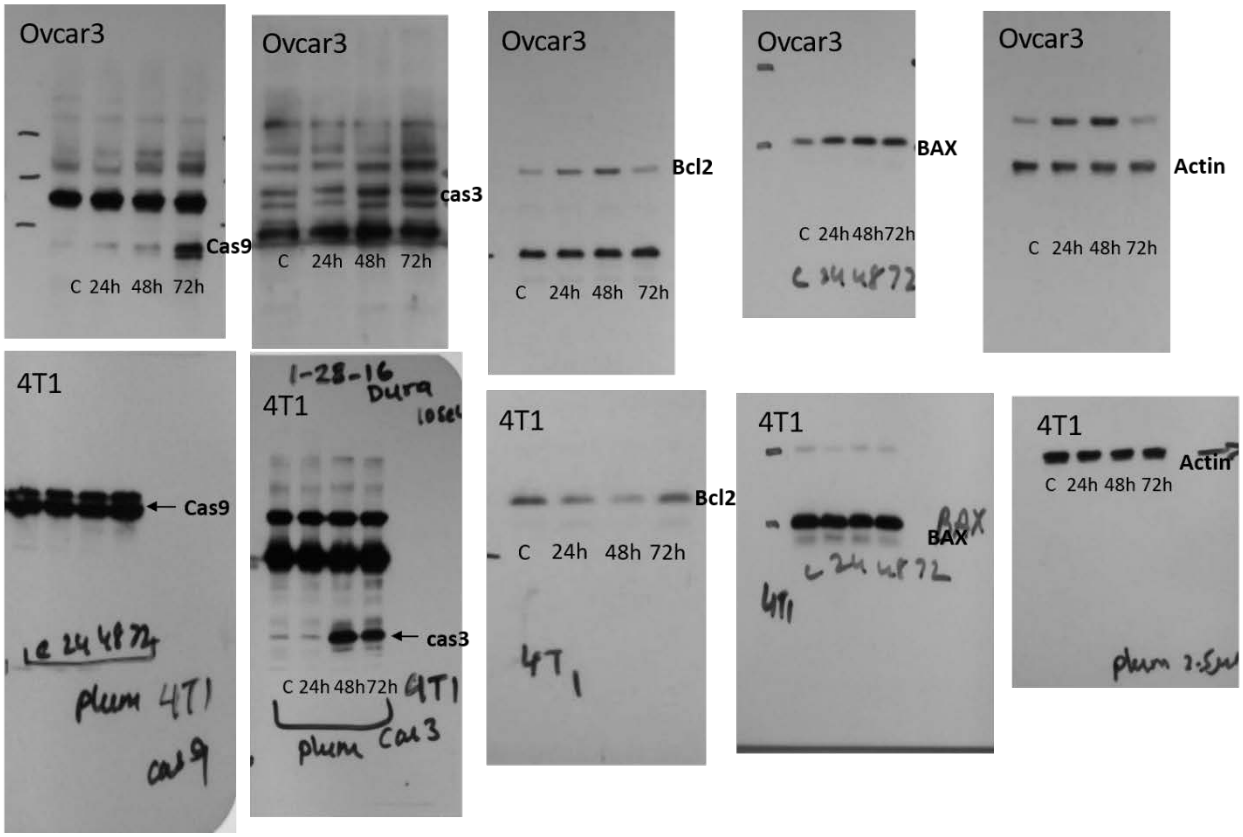

Supplementary File 10 – Full blots for Figure 8

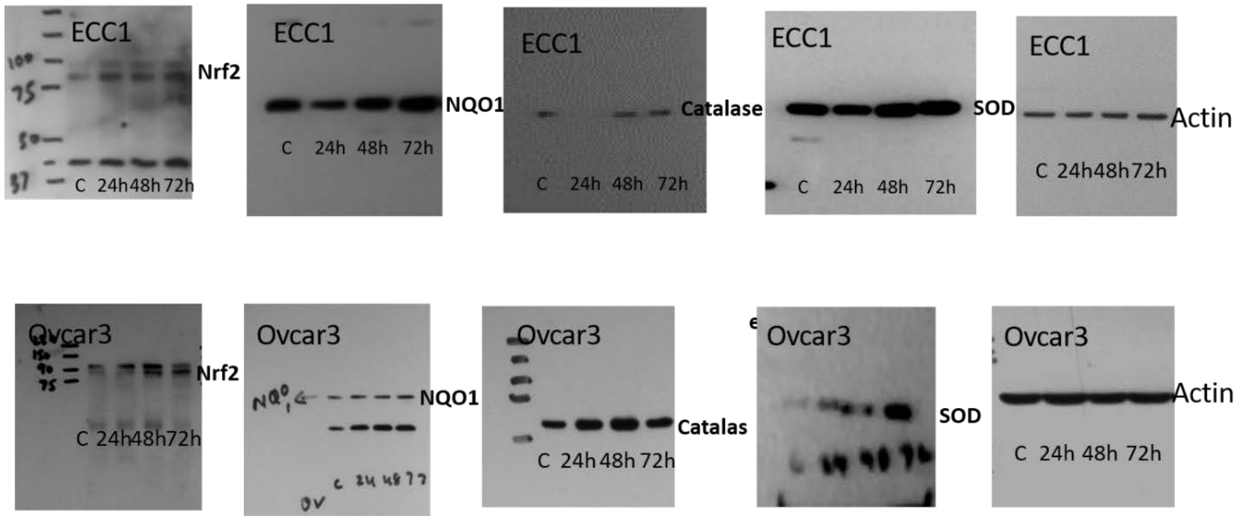

Supplementary File 10 – Full blots for Figure 8

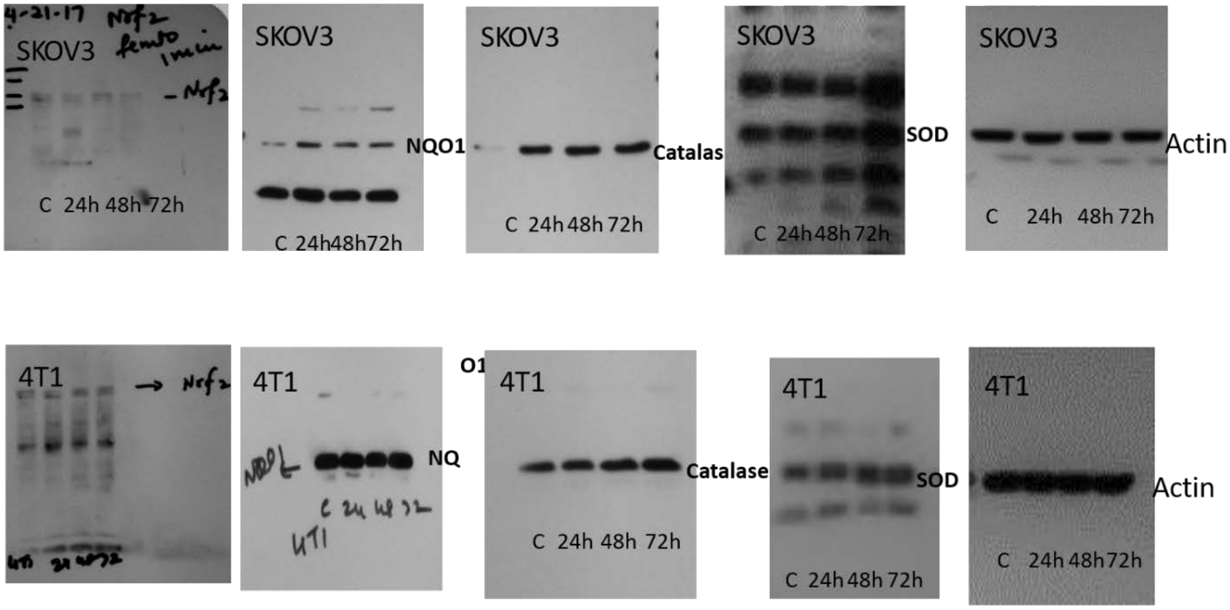

Supplement: Supplementary file 1 — Supporting data [file 41598_2018_19261_MOESM1_ESM.pdf]
